# Fungal and bacterial microbiome dysbiosis and imbalance of trans-kingdom network in asthma

**DOI:** 10.1186/s13601-020-00345-8

**Published:** 2020-10-22

**Authors:** Chunrong Huang, Youchao Yu, Wei Du, Yahui Liu, Ranran Dai, Wei Tang, Ping Wang, Chenhong Zhang, Guochao Shi

**Affiliations:** 1 Department of Pulmonary and Critical Care Medicine, Ruijin Hospital, Shanghai Jiao Tong University School of Medicine, 197, Rui Jin Er Road, Shanghai, 200025 People’s Republic of China; 2 Institute of Respiratory Diseases, Shanghai Jiao Tong University School of Medicine, 197, Rui Jin Er Road, Shanghai, 200025 People’s Republic of China; 3 State Key Laboratory of Microbial Metabolism and Ministry of Education Key Laboratory of Systems Biomedicine, School of Life Sciences and Biotechnology, Shanghai Jiao Tong University, 800, Dongchuan Road, Shanghai, 200240 People’s Republic of China

**Keywords:** Mycobiome, Bacteriome, Asthma, Correlations, Metagenomics

## Abstract

**Background:**

Fungal and bacterial microbiota play an important role in development of asthma. We aim to characterize airway microbiome (mycobiome, bacteriome) and functional genes in asthmatics and controls.

**Methods:**

Sputum microbiome of controls, untreated asthma patients and inhaled corticosteroid (ICS) receiving patients was detected using high throughput sequencing. Metagenomic sequencing was used to examine the functional genes of microbiome.

**Results:**

1. Mycobiome: α diversity was lower in untreated asthma group than that in controls. Mycobiome compositions differed among the three groups. Compared with controls, untreated asthma group has higher abundance of Wallemia, Mortierella and Fusarium. Compared with untreated asthma patients, ICS receiving patients has higher abundance of Fusarium and Mortierella, lower frequency of Wallemia, Alternaria and Aspergillus. 2. Bacteriome: α diversity was lower in untreated asthma group than that in controls. There are some overlaps of bacteriome compositions between controls and untreated asthma patients which were distinct from ICS receiving patients. Untreated asthma group has higher Streptococcus than controls. 3. Potential fungal and bacterial biomarkers of asthma: Trametes, Aspergillus, Streptococcus, Gemella, Neisseria, etc. 4. Correlation network: There are dense and homogenous correlations in controls but a dramatically unbalanced network in untreated asthma and ICS receiving patients, which suggested the existence of disease-specific inter-kingdom and intra-kingdom alterations. 5. Metagenomic analysis: functional pathways were associated with the status of asthma, microbiome and functional genes showed different correlations in different environment.

**Conclusion:**

We showed mycobiome and bacteriome dysbiosis in asthma featured by alterations in biodiversity, community composition, inter-kingdom and intra-kingdom network. We also observed several functional genes associated with asthma.

## Introduction

Asthma is a heterogeneous disease as a result of complex interactions between genetic and environmental factors [1], the definite mechanisms of susceptibility to certain clinicopathological features of the disease remain to be further delineated. Recently, two lines of evidence: dysbiosis of airway microbiome in the context of bacteria through 16S rRNA gene sequencing indicated in asthma patients [2, 3], and enhanced inflammatory responses in the absence of microbial colonization (germ free) in airways and intestinal tract in animal models [4, 5], drove researchers to propose the significant role of microbiome and its metabolic function in the incidence of asthma.

Healthy human airways, historically presumed as sterile, were recently reported to be composed of bacteria (predominant Proteobacteria, Firmicutes and Bacteroidetes), fungi and virus based on culture-independent molecular methods [6]. The paramount impact of microbiome on asthma could be implied by the early evidence of the development of asthma due to microbial colonization of distinct specific bacteria in the airways, such as Haemophilus, Moraxella, and Streptococcus [7–9]. Airway bacterial microbiota (bacteriome) was demonstrated to correlate with severity of airways obstruction, airway inflammation [10], bronchial hyperresponsiveness [11] and corticosteroid resistance [12] in asthma. However, until now, altered composition of airway fungal microbiota (mycobiome) in subjects suffering from asthma and its relation to clinical features is unclear. Except for the confirmation of significant abundance of Aspergillus in asthmatic patients and its positive association with impaired post-bronchodilator expiratory volume in 1 s based on conventional culture-method [13, 14], only Sharma et al. identified airway mycobiome in asthma, they explored the fungal diversity and features in endobronchial brush (EB) and bronchoalveolar lavage (BAL) samples from different inflammation phenotypes [15]. These results invited the speculation that, in addition to disturbance of bacteriome in unremittingly quiescent balanced lung microbiome, variations of community composition in mycobiome are also of vital importance in human susceptibility to asthma.

Here we sought to characterize airway microbiome (mycobiome, bacteriome) and their functional genes in healthy controls, untreated asthma patients and ICS receiving asthmatic patients, the study also aimed to identify bacterial-fungal correlations in these groups.

## Materials and methods

### Subjects

Chronic asthmatic patients were recruited from the Respiratory Outpatient Department, Ruijin Hospital, Shanghai Jiao Tong University School of Medicine, from October 2018 to July 2019. ICS is a standard treatment for asthma, we included both untreated asthma patients and ICS receiving patients. Diagnosis of asthma was established based on current episodic respiratory symptoms and evidence of variable airflow obstruction: forced expiratory volume in the first second (FEV1) improvement ≥ 12% and 200 mL after albuterol [16]. Individuals with acute attack, smoking history, diabetes, cancer, autoimmune diseases, and infectious diseases were excluded. Healthy control donors who were free of smoking, any respiratory symptoms and had normal lung function served as control subjects. All the subjects could be divided into three subgroups: Healthy controls (CON group), untreated asthma patients (untreated asthma group), patients receiving ICS (ICS asthma group). All the subjects were not treated with antibiotics or systemic glucocorticoids within 1 month before the study and they did not receive immunosuppressive agents or immunotherapy. We note that it’s difficult to induce sputum in some subjects, especially in healthy controls, these subjects were excluded, therefore, the sample size is relatively small.

The demographic data were recorded, including age, sex, body mass index (BMI), history of rhinosinusitis, smoking history, ICS dose, percentage of sputum eosinophils (EOS%) and neutrophils (NEU%), FEV1 percent predicted (FEV1% pre), FEV1/forced vital capacity percentage (FEV1/FVC), duration of asthma and Asthma Control Questionnaire 7 score (ACQ7 score).

### Ethics statement

The study was approved by the Ethics Committee of Ruijin Hospital (number: 2019-73). Written informed consent was obtained from individual subjects enrolled in this study after account of benefits and risks. All procedures were performed in accordance with the Declaration of Helsinki.

### Sample collection

Sputum was produced after induction by hypertonic saline nebulization, as previously described [17]. Samples for cell differential counts were stored at room temperature, those for DNA extraction were transported into sterile tubes and frozen immediately with dry ice and then stored at − 80 °C prior to analyses. Blood was collected and serum was stored at − 80 °C for IgE measurement.

#### Sputum processing

Briefly, the sputum was weighed and freshly prepared 0.1% solution of dithiothreitol (DTT) was added, then the mixture was vortexed for 15 min. After filtration through two layers of a sterile gauze the sputum was centrifuged for 10 min at 800 g. Supernatants were collected and stored at − 80 °C for interleukin measurements, including interleukin 4 (IL-4), IL-5, IL-6, tumor necrosis factor α (TNF-α).

### DNA extraction

DNA in sputum samples were extracted using the QIAamp DNA Microbiome Kit (Qiagen, Hilden, Germany, 51704) according to the manufacturer’s instructions, then DNA samples were stored at − 20 °C.

### Sequencing of 16S ribosomal RNA/internal transcribed spacer (16S rRNA/ITS) gene amplicon

The 16S rRNA and ITS genes were amplified and purified to prepare a library for sequencing at Majorbio (Shanghai, China) by using Illumina Miseq system. Operational Taxonomic Units (OTUs) was used to analyze sequencing data based on sequence similarity. An OTU is an organizational proxy of species produced by clustering using the UPARSE algorithm with > 97% sequence similarity [18]. In order to compare the community composition of each sample in each taxonomic level, each OTU was taxonomically classified against the SILVA and UNITE databases using RDP Classifier which provides taxonomic assignments from domain to genus. Details are provided in Additional file 1.

### Metagenomic sequencing

Extracted DNA was exposed to metagenomic sequencing. Functional annotations were performed by BLASTP against Kyoto Encyclopedia of Genes and Genomes (KEGG) databases to predict the gene function in corresponding pathways. Details on sequencing are provided in Additional file 1.

### Statistical analyses

α (difference within a sample) diversity included richness (Chao and Ace indices) and diversity (Shannon and Simpson indices, Phylogenetic diversity). Chao and Ace indices mean the total number of unique OTUs detected, Shannon and Simpson indices indicate the diversity and evenness (relative distribution) of OTUs in samples, Phylogenetic diversity, apart from diversity and evenness, it additionally accounts for phylogenetic relationships. Dimension reduction analysis by supervised sparse partial-least squares discriminant analysis (sPLS-DA) and Permutational Multivariate Analysis of Variance (PERMANOVA) testing was applied to figure out whether the microbiome composition was significantly different between different groups. Linear discriminant analysis (LDA) effect size (LEfSe) analysis, a method for biomarker discovery, was used to identify differentially abundant bacterial taxa or fungi that best characterize the populations of these groups. LDA score > 2 and P < 0.05 were considered to be significants. Random Forest (RF) classifier in R package (version. 3.6.2) was utilized to identify biomarker associated with asthma. Differences between groups were identified using the Kruskal–Wallis rank-sum test in R. Corrections were made using the False Discovery Rate multiple testing correction. Results were considered statistically significant for p-values ≤ 0.05. Details are provided in Additional file 1.

## Results

### Airway mycobiome

In this part, after filtering for low-quality reads and eliminating reads that did not match barcode sequences, specimens from 68 subjects were obtained for airway mycobiome evaluation, including CON (n = 16), untreated asthma (n = 22) and ICS asthma (n = 30) groups. Demographic characteristics of study subjects were summarized in Additional file 2: Table S1. As expected, people with rhinosinusitis are more likely to develop asthma, and significant differences in lung function were detected, as assessed by both FEV1% pre and FEV1/FVC. ICS asthma group had a longer duration of asthma than untreated asthma group (Additional file 2: Table S1).

### Taxonomic characterization of mycobiome

**Fig. 1:**
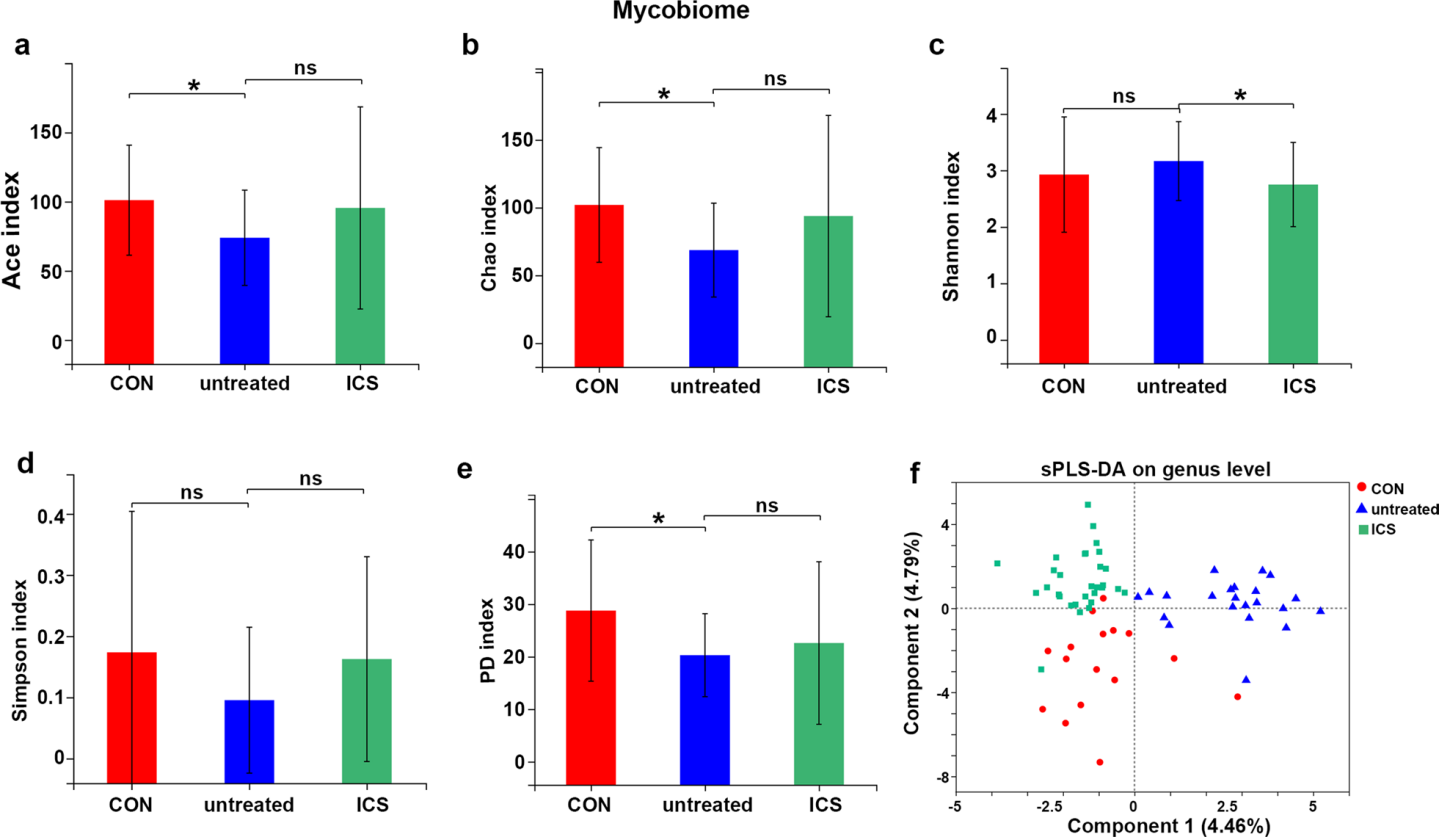
α and β diversity of the airway mycobiome. **a**, **b** Mycobiome richness as indicated by Ace and Chao indices. **c**–**e** Shannon, Simpson and PD indices. Statistical significance was determined using Kruskal–Wallis rank-sum test. ***p ≤ 0.001, **p ≤ 0.01, *p ≤ 0.05. **f** sPLS-DA multivariate analysis

We calculated the within-sample (α) diversity, Ace and Chao indices showed that richness of sputum mycobiome in untreated asthma group was significantly lower than that in CON group (Fig. 1a, b). The diversity (Shannon and Simpson indices) did not show any differences between these two groups, while Phylogenetic diversity (PD), weighting the phylogenetic relatedness of the fungi detected, showed less phylogenetically diverse fungal communities in untreated asthma group than that in CON group (Fig. 1c, e). However, analysis with a range of alternative α diversity indices (Ace, Chao, Shannon, Simpson and PD indices) resulted the only difference in Shannon index between untreated asthma and ICS asthma groups, the later had lower diversity compared with untreated asthma group (Fig. 1a–e).

There were positive correlations between FEV1%pre and Chao (r = 0.35, P < 0.01), Ace (r = 0.33, P < 0.01), and PD indices (r = 0.31, P = 0.01) (Additional file 3: Fig. S1). Chao index demonstrated an inverse relationship to ACQ7 score in asthmatic patients (r = − 0.28, P < 0.05) (Additional file 3: Fig S1). These results suggested that lower fungal richness in general is associated with worse lung function and worse asthma control.

Dimension reduction analysis by supervised sparse partial-least squares discriminant analysis (sPLS-DA) showed community compositions were clearly distinct among the three groups (PERMANOVA test P = 0.001) (Fig. 1f; Additional file 4: Table S2). At the phylum level, airway mycobiome among all groups exhibited predominant Ascomycota (66.17%), to a lesser extent Basidiomycota (21.48%), followed by unclassified_k__Fungi (9.16%) and Mortierellomycota (2.36%) (Additional file 5: Fig S2). At the genus level, the prevalent fungi were Candida (9.49%), unclassified_k__Fungi (9.16%), Wallemia (8.41%), Alternaria (8.34%), Aspergillus (5.7%), Cladosporium (5.56%) (Additional file 5: Fig S2).

**Fig. 2:**
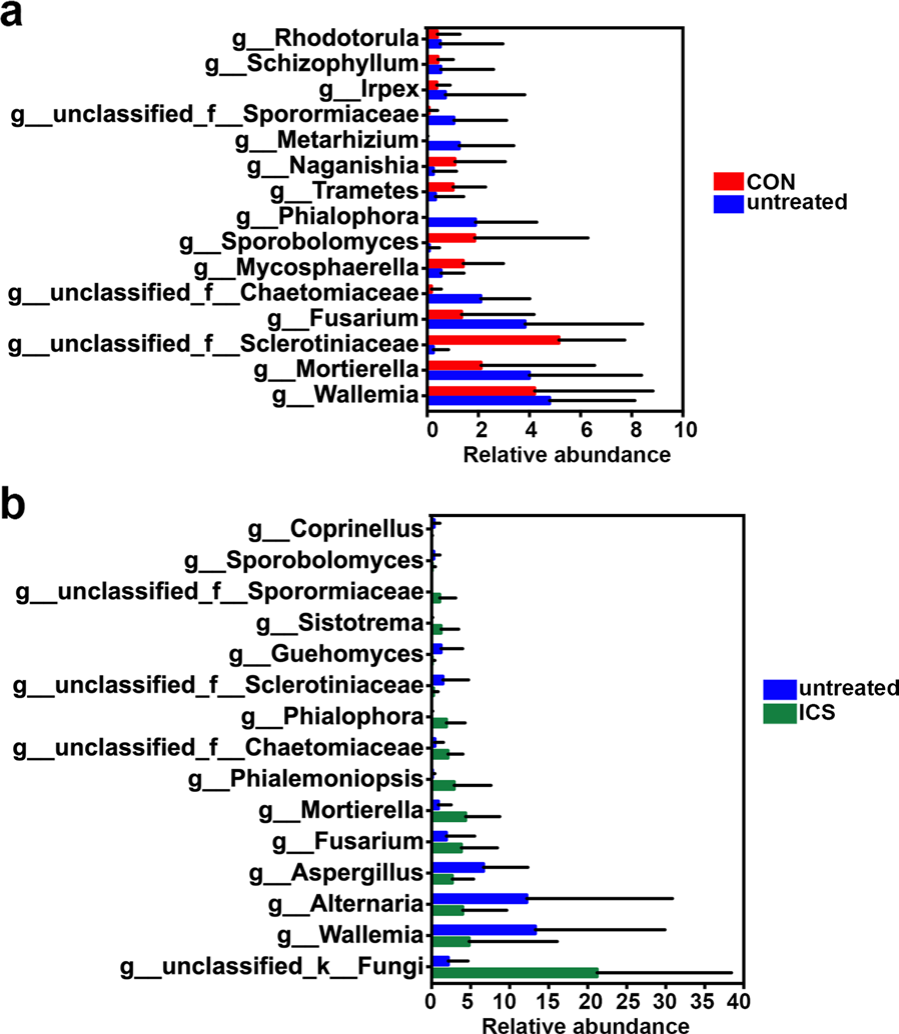
Relative abundance of top 15 genera in airway mycobiome. Significantly differing mycobiome between CON and untreated asthma group (**a**), between untreated asthma group and ICS asthma group (**b**) were shown. Statistical significance was determined using Kruskal–Wallis rank-sum test. All p < 0.05

Next we compared the relative abundances of genera between untreated asthma patients and controls. The top 15 significantly different genera were showed in Fig. 2: 10 genera were enriched in untreated asthma group, including Wallemia, Mortierella, Fusarium, unclassified_f_Chaetomiaceae, Phialophora, Metarhizium, unclassified_f_Sporormiaceae, Irpex, Schizophyllum, Rhodotorula, while 5 genera were reduced in untreated asthma group, including unclassified_f_Sclerotiniaceae, Mycosphaerella, Sporobolomyces, Trametes, Naganishia (Fig. 2a; Additional file 6: Table S3). To explore the effect of ICS on airway mycobiome community, we calculated the abundance of genera in ICS asthma group and untreated asthma group. Among the top 15 significantly different genera, 8 genera were increased in ICS asthma group, including unclassified_k_Fungi, Fusarium, Mortierella, Phialemoniopsis, unclassified_f_Chaetomiaceae, Phialophora, Sistotrema, unclassified_f_Sporormiaceae. By contrast, 7 genera were reduced in ICS asthma group, including Wallemia, Alternaria and Aspergillus, unclassified_f_Sclerotiniaceae, Guehomyces, Sporobolomyces, Coprinellus (Fig. 2b; Additional file 7: Table S4).

### Unique biomarkers detected in airway mycobiome

**Fig. 3:**
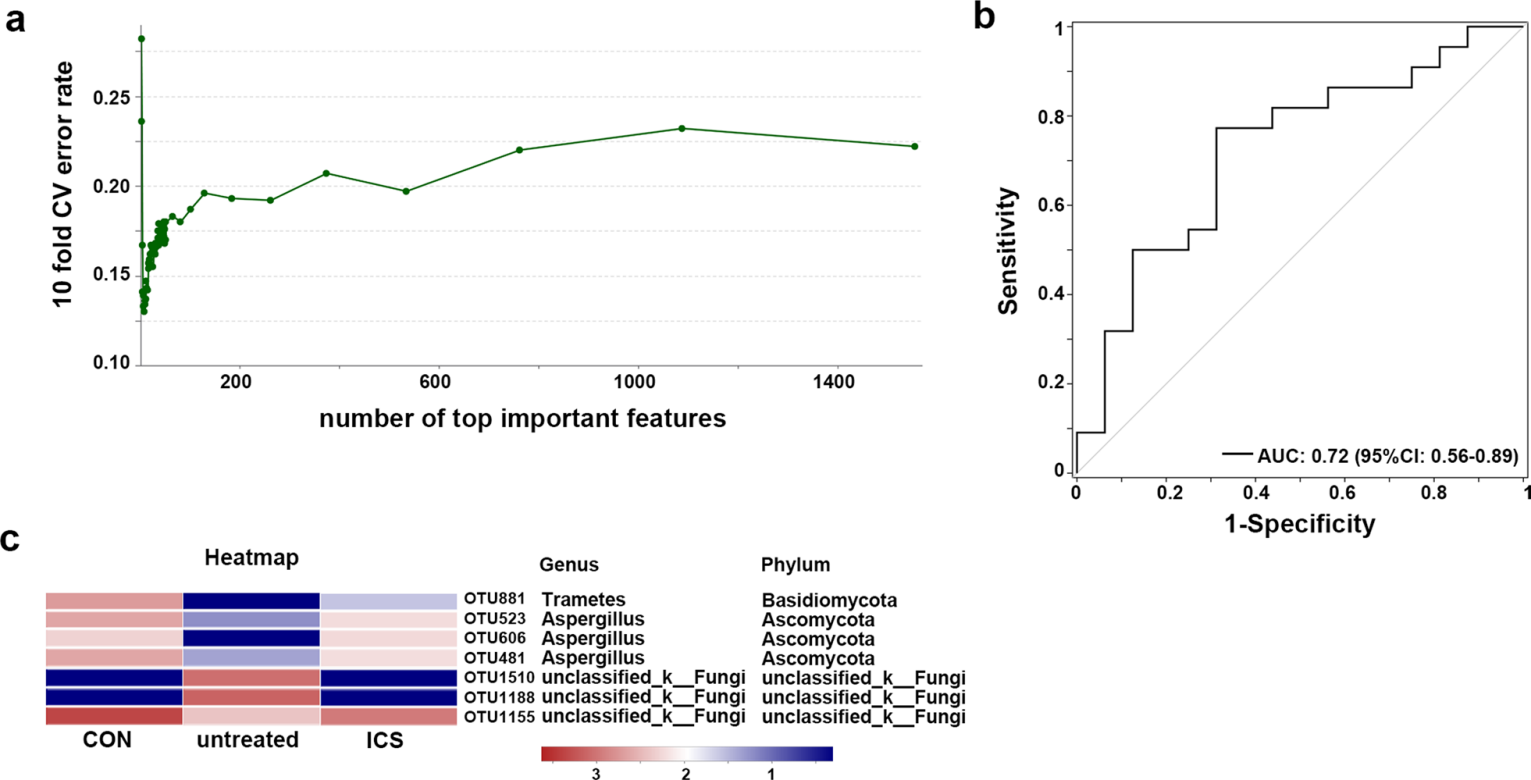
Biomarkers detected in airway mycobiome and comparisons of these biomarkers. **a** Prediction models using Random Forest (RF). X-axis represents the number of important species (variables) ranking top n, y-axis represents the corresponding prediction error rate using ten-fold cross validation (CV). **b** The Receiver Operating Characteristic (ROC) curve for the random forest model using the 7 OTUs. **c** The heat map showed the relative abundance of these biomarkers (mycobiome) in different groups

To further investigate the most important features more likely to explain the differences between the status of asthma and healthy conditions (untreated asthma vs CON), classification accuracy through Random Forest (RF) classifier with tenfold cross-validation was utilized to identify the features that contributed the most to the difference between CON and untreated asthma groups. Figure 3a depicts the top 7 important features associated with the classification when the error rate was the lowest: OTU1510 (g_unclassified_k_Fungi), OTU1188 (g_unclassified_k_Fungi), OTU1155 (g_unclassified_f_Sclerotiniaceae), OTU881 (g_Trametes), OTU606 (g_Aspergillus), OTU523 (g_Aspergillus), OTU481(g_Aspergillus). Using the 7 OTUs as microbiome markers to discriminate the untreated asthma group from the healthy control group, the area under the receiver operating characteristic curve (AUC) was 72%, and the 95% confidence interval (CI) was 56–89% (Fig. 3b). The abundance of OTU881 (g_Trametes), OTU523 (g_Aspergillus), OTU606 (g_Aspergillus), OTU481(g_Aspergillus) and OTU1155 (g_unclassified_f_Sclerotiniaceae) were lower in untreated asthma group than CON group, however, these OTUs increased after ICS medications (ICS asthma group) (Fig. 3c). The increased OTU1510 and OUT 1188 in untreated asthma group were reduced in ICS asthma group, similar levels to CON group (Fig. 3c). Together, these results demonstrated a trend for the variations of these taxa in untreated asthma patients to recover after ICS treatment (ICS asthma group).

### Airway bacteriome

82 induced sputum samples from CON (n = 26), untreated asthma (n = 23), and ICS asthma (n = 33) groups were obtained for 16S rRNA sequencing. Additional file 8: Table S5 showed that people with rhinosinusitis are more likely to develop asthma, untreated asthma group had lower FEV1% pre and FEV1/FVC compared with CON group. ICS asthma group had a longer disease duration and lower ACQ7 score than untreated asthma group (Additional file 8: Table S5).

### Taxonomic characterization of biome

**Fig. 4:**
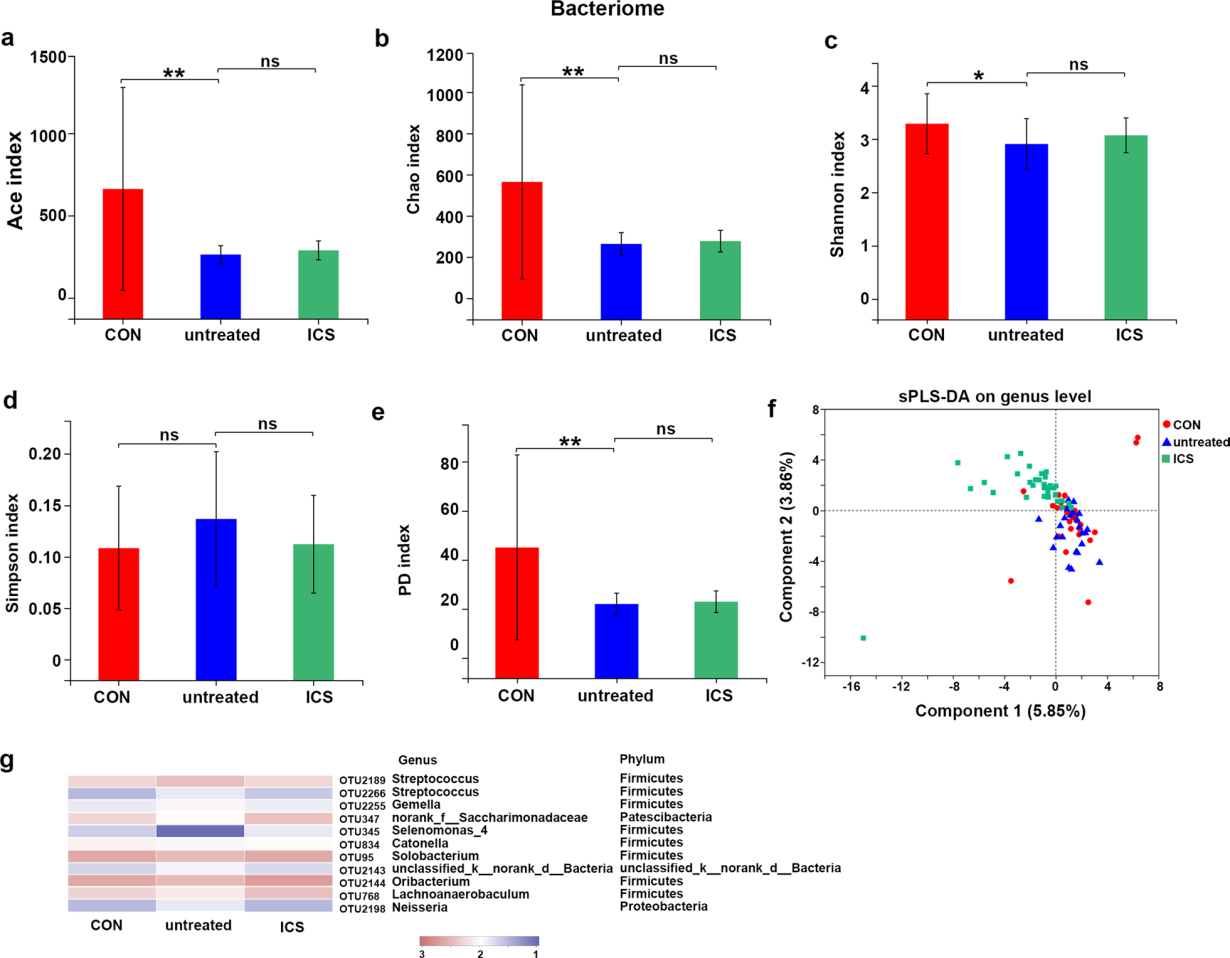
α and β diversity of the airway bacteriome. **a**, **b** Bacteriome richness as indicated by Ace and Chao indices. **c**–**e** Shannon, Simpson and PD indices in each group. Statistical significance was determined using Kruskal–Wallis rank-sum test. ***p ≤ 0.001, **p ≤ 0.01, *p ≤ 0.05. **f** sPLS-DA multivariate analysis. **g** The heat map showed the relative abundance of these biomarkers (bacteriome) identified by LEfSe in different groups

α diversity indices (Ace, Chao, Shannon and PD indices) indicated lower richness and less diverse array of bacteriome in untreated asthma group than CON group (Fig. 4a–e). However, no difference was showed in α diversity between untreated asthma and ICS asthma group (Fig. 4a–e).

sPLS-DA showed some overlaps of CON and untreated asthma groups which were distinct from the ICS asthma group (PERMANOVA test P = 0.01) (Fig. 4f, Additional file 9: Table S6). At the phylum level, bacteria from the phyla Firmicutes (44.9%), Actinobacteria (19.32%), Proteobacteria (16.37%) and Fusobacteria (9.26%) were predominant in all groups (Additional file 10: Fig S3). At the genus level, the top six bacteria were Streptococcus (27.26%), Rothia (12.6%), Neisseria (8.75%), Leptotrichia (6.02%), Actinomyces (5.54%), Haemophilus (5.10%) (Additional file 10: Fig S3).

Then we estimated top 15 genera significantly differing between CON group and untreated asthma group, the proportion of dominant Streptococcus was increased in untreated asthma group. When compared untreated asthma group with ICS asthma group, the relative abundances of 2 genera were enriched in ICS asthma group, while other 13 genera were reduced (Additional file 11: Fig S4; Additional files 12, 13: Table S7–S8).

### Discriminant bacteria detected in airway bacteriome

To illustrate the potential diagnostic value of key OTUs in airway bacteriome for the status of asthma, we also used RF in an attempt to detect untreated asthmatic samples, but it demonstrated a relatively low classification accuracy (AUC: 0.61, 95% CI 0.44–0.77) (Additional file 14: Fig S5a, b). Therefore, we used LEfSe to identify the discriminant taxa enriched in different groups (CON vs untreated asthma group, untreated asthma vs ICS asthma group) (Additional file 14: Fig S5c, d), the consistent OTUs from the two comparisons were presented in Fig. 4g. Compared with CON group, the abundance of OTU2189 (g_Streptococcus), OTU2266 (g_Streptococcus), OTU2255 (g_Gemella), OTU2198 (g_Neisseria) increased in untreated asthma group, however the proportions of these OTUs decreased in ICS asthma group. The abundance of OTU347 (g_norank_f_Saccharimonadaceae), OTU345 (g_Selenomonas_4), OTU834 (g_Catonella), OTU95 (g_Solobacterium), OTU2144 (g_Oribacterium), OTU768 (g_Lachnoanaerobaculum) were lower in untreated asthma group than that in CON group, which were further increased in ICS asthma group (Fig. 4g).

### Association between airway microbiome and clinical indices

**Fig. 5:**
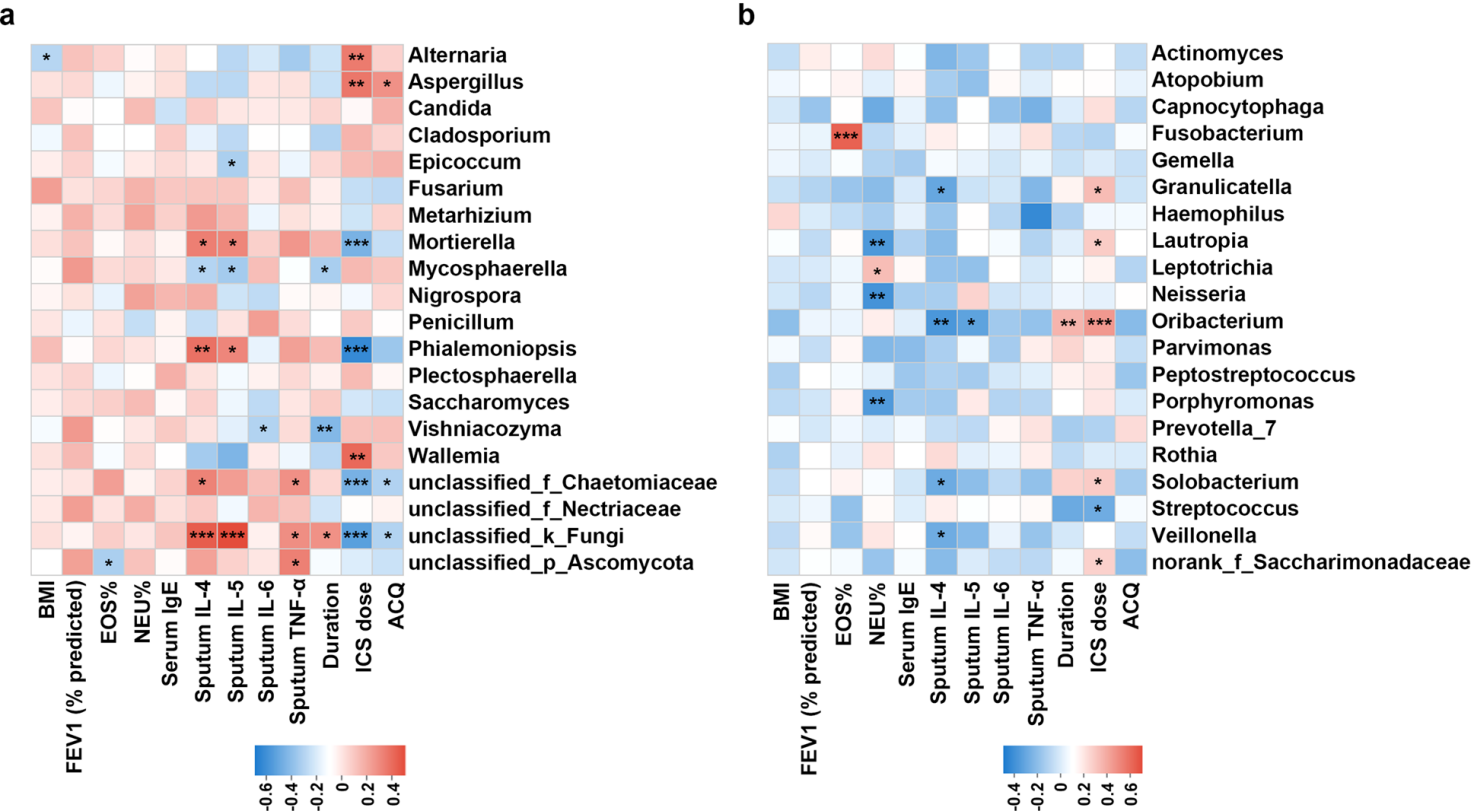
Correlation between airway mycobiome (**a**), bacteriome (**b**) and clinical indices of asthma. Correlations were performed based on Spearman correlation. Red indicates positive correlation; blue indicates negative correlation. ***p ≤ 0.001, **p ≤ 0.01, *p ≤ 0.05

We asked whether mycobiome and bacteriome correlated with clinical indices of asthma based on Spearman correlation analysis (Fig. 5). In mycobiome, genus Alternaria positively correlated with ICS dose, but negatively correlated with BMI. Aspergillus positively correlated with ICS dose and disease duration. Mortierella and Phialemoniopsis positively correlated with sputum IL-4 and IL-5 levels, while negatively correlated with ICS dose (Fig. 5a). In bacteriome, Fusobacterium positively correlated with EOS%, Neisseria negatively correlated with NEU%. Streptococcus negatively correlated with ICS dose (Fig. 5b).

### Fungal-bacterial correlation in airway microbiome

**Fig. 6:**
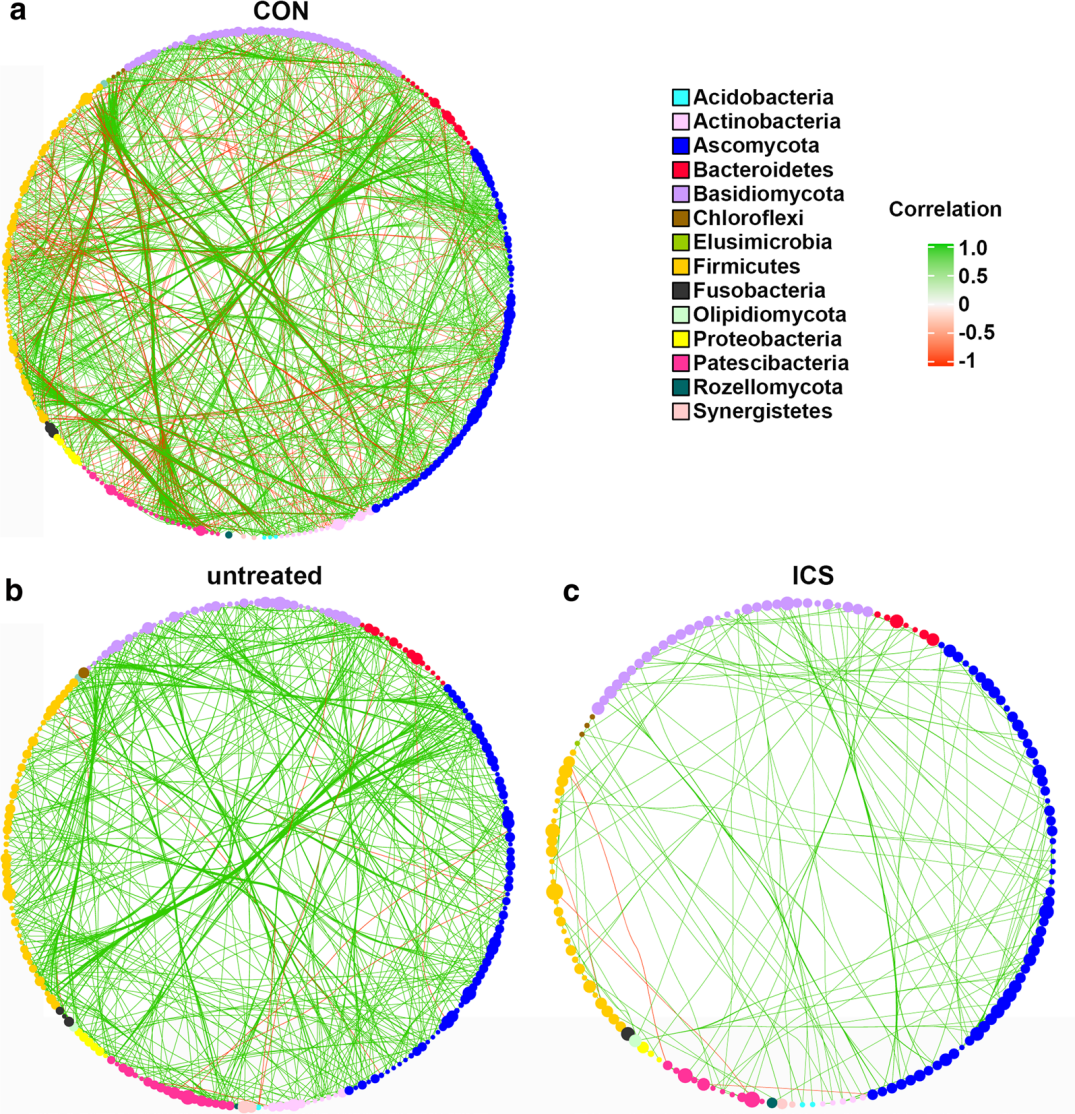
Fungal-bacterial network with top 150 genera. Networks in CON (**a**), untreated asthma (**b**) and ICS asthma (**c**) group were performed by using Cytoscape. Each circle (node) represents a microbial genus, its colour represents the bacterial or fungal phylum it belongs to and its size represents the number of direct edges that it has. The edge colour indicates the magnitude of the distance correlation; green indicates positive correlation and red indicates negative correlation (determined using spearman test). Only significant correlations (p value < 0.05 after false discovery rate correction) are displayed

To demonstrate inter-kingdom and intra-kingdom correlations in asthma, correlation networks derived from the airway microbiome dataset involving mycobiome or bacteriome at genus level were built to decipher the potential role of interaction patterns among different taxa in this study (Fig. 6). Samples from CON group had a greater density of connections between nodes compared with asthmatic samples. In CON group, mycobiome and bacteriome diversity was higher (Figs. 1 and 4), with a network showing positive and negative correlations distributed throughout the nodes. Both positive and negative correlations from Firmicutes to Basidiomycota and Proteobacteria, from Proteobacteria to Ascomycota were observed. Many positive correlations from Basidiomycota to Ascomycota were also observed. In addition, many genera within one phylum (Firmicutes, Basidiomycota and Ascomycota) connected with each other (Fig. 6a). Strikingly, networks in untreated asthma and ICS asthma group were dramatically different. Correlations above were decreased in untreated group. Notably, many negative correlations connecting genera from Firmicutes phylum to members of Basidiomycota and Proteobacteria phylum, from Proteobacteria phylum to Ascomycota phylum were decreased in untreated asthma group (Fig. 6). However, these correlations were much less in ICS asthma group relative to untreated group. There was a negative correlation between Firmicutes phylum and Proteobacteria phylum (Fig. 6c).

Taken together, these results suggest a complex relationship between the bacteria and fungi in airway microbiome and that specific alterations of connections in inter-kingdom and intra-kingdom are present in untreated asthma patients and ICS receiving patients.

### Comparison of inferred metagenomes

We used MicroPITA (microbiomes: Picking Interesting Taxonomic Abundance) [19] to select the most representative samples from previous subjects to perform metagenomic sequencing. A total of 15 samples were selected from CON (n = 5), untreated asthma group (n = 10) respectively for the use of sequencing. On level 1 (human disease), the abundance of genes related to asthma and Toxoplasmosis were higher in untreated asthma group than CON group (Additional file 15: Fig. S6a). On level 3, the abundance of genes related to Th1 and Th2 cell differentiation, Vitamin B6 metabolism were higher in untreated asthma group than CON group, contrastingly, the abundance of SNARE interactions in vesicular transport, transforming growth factor-beta (TGF-β) signaling pathway, Stilbenoid, diarylheptanoid and gingerol biosynthesis, Flavonoid biosynthesis, Fluorobenzoate degradation were lower in untreated asthma group than CON group (Additional file 15: Fig. S6b). Different members of airway microbiome were related to these pathways between CON and untreated asthma groups. Vitamin B6 metabolism were mainly associated with Rothia_mucilaginosa, other species also play a role such as Neisseria_meningitidis, Haemophilus_parainfluenzae Neisseria_flavescens and Actinpyyces_sp._ICM47. Other functional genes were mainly related with unclassified species (Additional file 16: Fig S7).

### Correlations between microbiome and functional genes

Then, to demonstrate the global relationships between microbiome and functional genes, we built correlation networks at genus level involving the top 20 taxa and top 30 functional genes in (Additional file 17: Fig S8). CON group consisted of mainly two clusters, in cluster 1, species from Bacteroidetes, Proteobacteria and Firmicutes showed less correlations with metabolism than untreated asthma group, species Rothia_mucilaginosa (phylum Actinobacteria) showed many correlations with several genes from metabolism and genetic information processing, including glycolysis/gluconeogenesis. However, untreated asthma group mainly consisted of a large cluster, species Rothia_mucilaginosa was associated with propanote metabolism and genes from environmental information processing (Additional file 17: Fig S8).

## Discussion

In this study, we observed airway mycobiome and bacteriome dysbiosis and inter-kingdom imbalance in asthmatic patients. The results also showed alterations of several functional genes associated with asthma, asthmatic patients also showed different correlations between microbiome and functional genes.

Research on airway bacterial microbiota (bacteriome) have been performed in recent years, there were no absolutely consistent results about the changes of microbial diversity and composition in asthma. For instance, we observed remarkably reduced bacterial diversity and richness in untreated asthma group when compared with CON group, this is partly inconsistent with previous studies, in which increased [11, 20, 21] or similar [12, 22] bacterial diversity or richness were reported in asthmatic patients. The frequency of the confirmed top five phyla also varied across studies [2, 12, 20, 23], even in the same populations from which samples were obtained, such as the comparison between our study (dominated by Firmicutes; roughly similar proportions of Actinobacteria and Proteobacteria; Bacteroidetes being the lowest) and Li’s study (roughly equal proportions of Firmicutes, Proteobacteria, and Bacteroidetes accounting for over 80%, Actinobacteria being the lowest) [23]. If we reflect on all the proposed factors, including sample size, sample sites, sample population, methods of measurement, different phenotypes of asthma, different medications and so on, it is not unexpected to find the interindividual heterogeneity of microbial composition in all human niches, including the lung.

Streptococcus is one of the common pathogens related to the development of asthma [8, 9], it is negatively associated with FEV1% predicted in asthmatics [24]. In our study, significantly increased abundance of Streptococcus was detected in untreated asthma patients, it was also one of the potential biomarkers of asthma in our study. We found OTUs belonging to potential pathogens such as genera Streptococcus, Gemella and Neisseria were discriminant enriched in untreated asthma group, these genera were reported to be positively correlated with eosinophil percentage [25]. Moreover, the proportions of these taxa decreased in ICS receiving asthma patients, indicating a trend for the bacteriome to recover after ICS treatment and suggesting the idea that airway microbiome can be affected by ICS prescription.

The link between fungi and asthma has been known for several decades, but the airway fungal microbiota (‘mycobiome’) is a new and emerging field lagging behind our understanding of bacteriome. Therefore, barely literature is available regarding the specific changes in airway mycobiome in asthma. We identified 4 major fungal phyla and 6 major fungal genera, and the findings of lower α diversity in asthma patients, positive correlations between α diversity metrics and improving lung function and asthma control, echoed a recent observation in subjects with T2-high asthma of low bronchial fungal diversity and their correlations with clinical parameters, such as FEV1, fraction of exhaled nitric oxide (FENO) values [15]. We also demonstrated reduced Shannon index of mycobiome in ICS asthma group compared with untreated asthma group, indicating lower diversity of airway mycobiome with the treatment of ICS. Significant taxonomic differences in mycobiome were detected between untreated asthma group and controls, 10 genera were enriched in untreated asthma group relative to CON group: Wallemia, Mortierella, Fusarium, unclassified_f_Chaetomiaceae, Phialophora, Metarhizium, unclassified_f_Sporormiaceae, Irpex, Schizophyllum, Rhodotorula. This is consistent with pioneering research on several of them, which has been suggested to be associated with asthma [15, 26]. We also noted reductions of well-known pathogens (Wallemia, Alternaria and Aspergillus) in ICS asthma group relative to untreated asthma group, supporting the idea that the ICS treatment can influence airway mycobiome.

We identified key OTUs belonging to genera Trametes, Aspergillus and unclassified_f_Sclerotiniaceae, to predict the presence of the status of asthma. 3 OUTs from genera Aspergillus were reduced in untreated asthma patients. Aspergillus was a strong candidate to be associated with disease severity or hospitalizations in individuals stricken with asthma and colonization of the airways by Aspergillus species correlated with severe asthma and nasal allergies [27, 28]. We noted that the 16S rRNA sequencing cannot denote the specific species and strains of Aspergillus, and the ability to produce toxin and pathogenicity varied from species, so this inconsistency of present study with clinical perspectives should be interpreted with caution and deserve further researches in the light of the 16S rRNA sequencing to better delineate their associations to clinical several limitations. Apart from that, our results also showed changes of these OTUs (untreated asthma group vs CON group) tend to reverse in ICS asthma group, indicating a trend for the variations of these taxa in untreated asthma patients to recover after ICS treatment (ICS asthma group). All in all, the proposal of the effect of ICS on microbiome can be dated back to prior authors who demonstrated an increase in Proteobacteria and Pseudomonas, a reduction in Bacteroidetes, Fusobacteria, and Prevotella species in those taking corticosteroid treatment, particularly the combination of ICS and oral corticosteroid (OCS) [29], and who reported that species from genus Penicillium enriched in BALF samples from asthmatic patients receiving ICSs [15].

Fungi and bacteria coexisted within the airway, commensal fungi and bacteria could be found together in healthy conditions, and the break of microbial equilibrium may be a cause of asthma. The speculations of fungal–fungal, bacterial–bacterial, and even fungal-bacterial interactions may be a missing link. It has been suggested in animal model that antibiotic treatment leads to major fungi expansions which were then reduced following antibiotic cessation [30], in return, antifungal treatment induced a change in overall bacterial community structure in gut [31]. These results indicated a balance between mycobiome and bacteriome in certain environment. We performed network analysis to observe the airway microbiome equilibrium, the results showed tighter interactions between fungi and bacteria in CON group, but the number and the intensity of the correlations were decreased in untreated asthma and ICS asthma groups, indicating less correlations between the two kingdoms. Therefore, asthma may be characterized by disrupted connections between mycobiome and bacteriome, and ICS treatment could not reverse the microbial biodiversity and inter-kingdom correlations. Further studies are needed to elucidate more precisely the inter-kingdom correlations in airway microbiome.

The results of metagenomic sequencing indicated that the status of asthma was mainly associated with functional genes from infectious diseases, immune disease, metabolism of cofactors and vitamins, xenobiotics biodegradation and metabolism, biosynthesis of other secondary metabolites, folding, sorting and degradation, immune system. For example, the abundance of genes related to asthma, Th1 and Th2 cell differentiation, Vitamin B6 metabolism were higher in untreated asthma group than CON group. Th1 and Th2 have been demonstrated to correlated with the features of asthma such as airways hyperreactivity, eosinophilic and neutrophilic inflammation, and airway remodeling, playing a role in the development of asthma [32]. Vitamin B6 can regulate productions of metabolites with immune regulatory roles, so changes of vitamin B6 metabolism can result in different diseases, including inflammatory disease [33]. However, fewer genes related to TGF-beta signaling pathway, Stilbenoid, diarylheptanoid and gingerol biosynthesis, Flavonoid biosynthesis were detected in untreated group than CON group. TGF-beta is important in regulating T cell homeostasis, airway remodeling, airway inflammation and apoptosis of airway smooth muscle cell, many studies demonstrated targeting at TGF-beta signaling showed therapeutic benefits for asthma to some extent [34–37]. Some bioactive compounds in ginger, such as gingerols, diarylheptanoids, and flavonoids, are of significant importance in human disease because of their anti-allergic and anti-inflammatory properties [38]. Flavonoid can also decrease serum IgE level, inhibit eosinophil inflammation and the release of Th2-associated cytokines in asthmatic animal models [39, 40]. Apart from these pathways, we also noted Toll-like receptor4 (TLR4), one of the major TLRs involved in the recognition of major bacterial components, plays an important role in targeting T cells response and the development of asthma. Some studies demonstrated beneficial effects of TLR4 agonists such as lipopolysaccharide (LPS) in asthma [41, 42], whereas others reported pivotal role of LPS-TLR4 signaling in asthma exacerbation, which paralleled clinical signs of microbial asthma exacerbation, including extended disease duration and worsen symptoms [43]. Thus, contribution of LPS-TLR4 signaling in asthma and the effect of ICS on it is also worthy of further investigation. Different species act different functional roles in the airway microenvironments because they possess different functional genes. For instance, Rothia_mucilaginosa contributed more to vitamin B6 metabolism than other species, interestingly, it demonstrated different correlation patterns with functional genes in healthy controls and asthma patients. Therefore, dysbiosis of airway microbiome in asthma was accompanied by alterations of connections between microbiome and functional pathways associated with the pathophysiology of asthma.

There are some limitations in our study. The relatively small sample size in our study may weaken the strength in some comparisons. The cross-sectional nature of our study and the exclusive selection of adult patients did not allow us to track the causal relationship between alterations in microbial community and asthma, which warranted to further focus. We also note the absence of patients taking OCS which can be used for further analysis of the effects of corticosteroid on microbiome in asthma. Likewise, the influence of air pollution should also be taken into consideration in the subsequent studies, because it has been reported that air pollution affects the distribution of microbiome communities in respiratory tract [44–46], especially in heavily air polluted areas. Still, oral contamination cannot be completely excluded in the process of sputum collection although the analysis of airway bacterial composition here yielded quite distinct results compared with oral bacteriome [47]. It is also important to note that relatively small number of samples analyzed in metagenomic sequencing may limit the accuracy of extrapolating the results obtained in real world. Finally, it’s difficult for us to recruit steroid resistant asthmatics because of the small subpopulation. We propose that microbiome plays a vital role in steroid-based therapies. Our data showed increased abundance of genus Streptococcus, the colonization of which has been reported to be positively associated with sputum IL-8 concentration and neutrophil count [48]. We also reported ICS treatment could decrease the abundance of genera Aspergillus, Alternaria, both of which were described in severe asthma with fungal sensitization (SAFS). However, the role of mycobiome and bacteriome in steroid resistant asthmatics is still remained to be delineated.

## Conclusion

In conclusion, we demonstrated simultaneously mycobiome and bacteriome dysbiosis in asthma characterized by alterations in diversity and composition. We revealed specific inter-kingdom between fungi and bacteria in asthma, as well as the alterations of functional genes. Future greater studies designed to elucidate causality and microbial mechanisms contributing to the pathophysiology of asthma may therefore benefit from longitudinal studies. The correlations between fungi and bacteria, between specific microbiome and functional genes are also further research avenues to pursue.

## Supplementary information

**Additional file 1:** Supplementary materials.

**Additional file 2: Table S1.** Demographic and clinical characteristics of study subjects (mycobiome) (n=68).

**Additional file 3: Fig. S1.** Relationships between variability in airway mycobiome composition and community diversity. a and b. Increased richness (higher Aceand Chao indices) is correlated with higher FEV1%pre. c. Increased phylogenetic diversity (higher PD index) is correlated with higher FEV1%pre.d. Increased richness (higher Chao index) is correlated with lower ACQ 7 score.

**Additional file 4: Table S2.** PERMANOVA of mycobiome community composition in sputum based on Bray-Curtis distance.

**Additional file 5: Fig. S2.** Circular representation of mycobiome communities visualized by Circos software. The outmost circles list names of three groups and mycobiome community composition. The length of the bars on the second ring represented the percentage of phyla or genera in three groups (left side of the diagram), and the percentage of sample in phyla or genus. The connecting lines inside the third circle link the phylum or genus to the groups and the width of the line indicates the relative abundance. The outset cycles were coloured according to the software default setting.

**Additional file 6: Table S3.** Relative abundance of top 15 genera in airway mycobiome differing significantly between CON and untreated asthma group.

**Additional file 7: Table S4.** Relative abundance of top 15 genera in airway mycobiome differing significantly between untreated asthma and ICS asthma group.

**Additional file 8: Table S5.** Demographic and clinical characteristics of study subjects (bacteriome) (n=82)

**Additional file 9: Table S6.** PERMANOVA of bacteriome community composition in sputum based on Bray-Curtis distance.

**Additional file 10: Fig. S3.** Circular representation of bacteriome communities in CON, naïve asthma and ICS asthma groups at phylum and genus level, visualized by Circos software. The outmost circles list names of three groups and bacteriome community composition. The length of the bars on the secondring represented the percentage of phyla or genera in three groups (left side of the diagram), and the percentage of sample in phyla or genus. The connecting lines inside the third circle link the phylum or genus to the groups and the width of the line indicates the relative abundance. The outset cycles were coloured according to the software default setting.

**Additional file 11: Fig. S4.** Relative abundance of top 15 genera in airway bacteriome differing significantly between CON and untreated asthma group (a), between untreated asthma group and ICS asthma group (b). Statistical significance was determined using Kruskal-Wallis rank-sum test.

**Additional file 12: Table S7.** Relative abundance of top 15 genera in airway bacteriome differing significantly between CON and untreated asthma group.

**Additional file 13: Table S8.** Relative abundance of top 15 genera in airway bacteriome differing significantly between untreated asthma and ICS asthma groups.

**Additional file 14: Fig. S5.** Prediction models and discriminant taxa of bacteriome. a. Prediction models using Random Forest (RF). X-axis represents the number of important species (variables) ranking top n, y-axis represents the corresponding prediction error rate using 10-fold cross validation (CV). b. The Receiver Operating Characteristic (ROC) curve for the random forest model. c,d. LEfSe analysis showing the differentially abundant taxonomy(CON vs untreated asthma group, untreated asthma group vs ICS asthma group). The LDA scores (log10) > 2 are listed, p ≤ 0.05.

**Additional file 15: Fig. S6.** Comparisons of functional genes between CON and naïve asthma group. a. Functional genes of level 1 (human disease) with significant differences between CON and untreated asthma group were showed (Kruskal-Wallis rank-sum test), corrections were made using the False Discovery Rate multiple testing correction. Results were considered statistically significant for p-values ≤ 0.05. b. Functional genes of level 3 with significant differences between CON and untreated asthma group were showed (Kruskal-Wallis rank-sum test), corrections were made using the False Discovery Rate multiple testing correction. Results were considered statistically significant for p-values ≤ 0.05.

**Additional file 16: Fig. S7.** Contributed species to Vitamin B6 metabolism.

**Additional file 17: Fig. S8.** Correlation network between microbiome and functional genes in CON (a) and untreated (b) group. The node represented a microbial genus, its colour represents the bacterial phylum it belongs to and its size represents the number of direct edges that it has. The green edges indicated positive correlation and red edges indicated negative correlation (Spearman test). Only significant correlations (p value < 0.05 after false discovery rate correction) are displayed.

**Additional file 18: Fig. S9.** Rarefaction curves of Shannon (a) and Simpson (b) indices of 68 samples (mycobiome). X: number of sequences per sample, Y: rarefaction measure.

**Additional file 19: Fig. S10.** Clusters of all the 52 asthmatic patients (mycobiome). a. The optimal number of types was five as indicated by the maximum CH index.b. The airway mycobiome of untreated asthma and ICS asthma groups are clustered into five types at the genus level, dominated by Wallemia (type 1), Candida (type 2), Alternaria (type 3), Plectosphaerella (type 4), and unclassified_k_Fungi (type 5), respectively. c. Relative abundancesof the top 10 genera in the five types (all p < 0.05). d. Distribution of the samples of the two groups in the five types.

**Additional file 20: Fig. S11.** Rarefaction curves of Shannon (a) and Simpson (b) indices of 82 samples (bacteriome). X: number of sequences per sample, Y: rarefaction measure.

**Additional file 21: Fig. S12.** Clusters of all the 56 asthmatic patients (bacteriome). a. The maximum CH index at two types indicated the optimal enterotype number. b. The airway bacteriome of untreated asthma and ICS asthma groups are clustered into two types at the genus level, dominated by either Streptococcus (type 1) or Neisseria (type 2). c. Relative abundances of the top 10 genera in the two types. The comparisons of Streptococcus, Neisseria, Haemophilus, Porphyromonas and Gemella were statistically different (p < 0.05). d. Distribution of the samples of the four groups in the two types.

## Data Availability

The data that supports the findings of this study are available in the Additional files of this article.

## Abbreviations

ICS: Inhaled corticosteroid
BMI: Body mass index
FEV1%pre: FEV1 percent predicted
FEV1/FVC: FEV1/forced vital capacity percentage
ACQ7 score: Asthma Control Questionnaire 7 score
16S rRNA: 16S ribosomal RNA
ITS: Internal transcribed spacer
OTUs: Operational Taxonomic Units
ORFs: Open reading frames
KEGG: Kyoto Encyclopedia of Genes and Genomes
PD: Phylogenetic diversity
PLS-DA: Dimension reduction analysis by supervised sparse partial-least squares discriminant analysis
PERMANOVA: Permutational Multivariate Analysis of Variance
LEfSe: Linear discriminant analysis (LDA) effect size
RF: Random Forest
AUC: Area under the receiver operating characteristic curve
MicroPITA: Microbiomes Picking Interesting Taxonomic Abundance
FENO: Fraction of exhaled nitric oxide
OCS: Oral corticosteroid
TGF-β: Transforming growth factor-beta
TLR4: Toll-like receptor4
LPS: Lipopolysaccharide
