# Supplementary material for: Fungal and bacterial microbiome dysbiosis and imbalance of trans-kingdom network in asthma
Source: Clin Transl Allergy. 2020 Oct 22;10:42. doi: 10.1186/s13601-020-00345-8 (PMC7583303; doi:10.1186/s13601-020-00345-8)
Supplement: Supplementary file 1 — Additional file 1: Supplementary materials. [file 13601_2020_345_MOESM1_ESM.pdf]

Additional 1: Supplementary materials

Supplementary Methods

Sequencing of 16S ribosomal RNA/internal transcribed spacer (16S rRNA/ITS) gene amplicon

The 16S rRNA and ITS genes were amplified and purified to prepare a library for sequencing at Majorbio (Shanghai, China) by using Illumina Miseq system. DNA was subjected to amplification of polymerase chain reaction (PCR) using primers directed at hypervariable region 3-4 (V3-V4) of the 16S rRNA gene (338F: 5'- ACTCCTACGGGAGGCAGCAG-3'; 806R: 5'-GGACTACHVGGGTWTCTAAT-3') and ITS (1F: 5'-CTTGGTCATTTAGAGGAAGTAA-3'; 2R: 5'-GCTGCGTTCTTCATCGATGC-3'). Each forward primer had an adaptor sequence and a barcode sequence to help identify the samples after sequencing. The sequences were analyzed using the Quantitative Insights Into Microbial Ecology (QIIME, version 1.9.1, http://qiime.org/install/index.html) software package. Fastp (version 0.19.6, https://github.com/OpenGene/fastp) was used for quality control with the following criteria: (i) The reads were truncated at any site receiving an average quality score <20 over a 50 bp sliding window. (ii) Reads containing ambiguous bases were discarded. (iii) Sequences whose overlap longer than 10 bp were merged according to their overlap sequence. Paired-end sequences were combined using Flash (version 1.2.11, https://ccb.jhu.edu/software/FLASH/index.shtml). Sequences were then assigned to Operational Taxonomic Units (OTUs) using the UPARSE algorithm with a 97% threshold pairwise identity and

taxonomically classified against the SILVA (version 132, https://www.arb-silva.de/) and UNITE (version 8, https://unite.ut.ee/) database by RDP Classifier algorithm [1].

Metagenomic sequencing

The metagenomic sequencing of extracted DNA was performed at Majorbio (Shanghai, China) by using Illumina Hiseq system. Libraries were constructed with insert size (around 325 bp) followed by high-throughput sequencing.

Data quality control. Seqprep (https://github.com/jstjohn/SeqPrep) was employed to remove the adapter sequences. Sickle (Version 1.33, https://github.com/najoshi/sickle) was used to discard the reads from the 3′ end until reaching the first nucleotide with a quality threshold of 20, remove read pairs shorter than 60 bp or containing "N" bases. Reads belonging to the host were also removed. A total of 262,406,348 high-quality reads with an average of 17,493,756.53 reads per sample were obtained.

De novo assembly and construction of the gene catalog. Further, these high-quality reads were assembled to contigs longer than 300 bp by using Megahit (Version 1,1,2, https://github.com/voutcn/megahit). Average 874036 contigs per sample with an average length of 595507001.7 bp were obtained. N50 was between 468 and 3206 bp. MetaGene was used to predict the open reading frames (ORFs) in obtained contigs. ORFs less than 100bp were filtered out, CD-HIT (http://www.bioinformatics.org/cd-hit/) with 95% identity and 90% coverage was employed to generate final

gene catalogue with total 1,408,637 non-redundant genes with an average length of 538.13 bp. High quality reads were then mapped onto the gene catalogue using SOAPaligner (http://soap.genomics.org.cn/soapaligner.html).

Finally, functional annotations were performed by BLASTP (BLAST Version 2.2.28+, http://blast.ncbi.nlm.nih.gov/Blast.cgi) against Kyoto Encyclopedia of Genes and Genomes (KEGG) databases to predict the gene function in corresponding functional pathways at a cut- off of E-value $< 10^{-5}$[2]. The abundance of genes was estimated by Reads Per Kilobase per Million mapped reads (RPKM).

Statistical analyses

The rarefied OTU table was used for all the subsequent analyses using QIIME (version 1.9.1). α (difference within a sample) diversity included richness (Chao and Ace indices) and diversity (Shannon and Simpson indices, Phylogenetic diversity). Chao and Ace indices mean the total number of unique OTUs detected, Shannon and Simpson indices indicate the diversity and evenness (relative distribution) of OTUs in samples, Phylogenetic diversity, apart from diversity and evenness, it additionally accounts for phylogenetic relationships. Dimension reduction analysis by supervised sparse partial-least squares discriminant analysis (sPLS-DA) and Permutational Multivariate Analysis of Variance (PERMANOVA) testing was applied to figure out whether the microbiome composition was significantly different between different groups. Microbial phyla and genera in three groups were visualized using Circos [3]. We also clustered all asthmatic patients using Jensen-Shannon Distance (JSD) and the

Partitioning around medoids (PAM) clustering algorithm, calculated the optimal number of clusters by Calinski-Harabasz (CH) index, and finally visualized the taxonomic drivers of clusters with Principal component analysis (PCA) [4]. Linear discriminant analysis (LDA) effect size (LEfSe) analysis, a method for biomarker discovery, was used to identify differentially abundant bacterial taxa or fungi that best characterize the populations of these groups. LDA score >2 and $P<0.05$ were considered to be significant Random Forest (RF) classifier in R package (version. 3.6.2) was utilized to identify biomarker associated with asthma. The correlation between α diversity and lung function and ACQ score were analyzed using linear regression in Prism (version. 7). The open source MicroPITA (microbiomes: Picking Interesting Taxonomic Abundance) (http://huttenhower.sph.harvard.edu/micropita) was used to select the most representative samples from previous subjects to perform metagenomic sequencing [5]. Differences between groups were identified using the Kruskal-Wallis rank-sum test in R. Corrections were made using the False Discovery Rate multiple testing correction. Correlation network between microbiome and functional genes was calculated using Networkx (version.2.4, http://networkx.github.io) (Spearman correlation). Results were considered statistically significant for p-values ≤ 0.05.

Supplementary results:

Airway mycobiome

4,222,025 amplicon sequence reads (average sequence length, 235.5 bp) were yielded after quality control, 12 phyla, 240 family, 452 genera, 710 species and 1553 OTUs were identified for further analysis. Rarefaction curves (Shannon and Simpson) of all samples on OTU level reached a plateau, indicating the sequencing data is large enough to reflect the majority of microbial information in all samples (Additional file 17: Fig S9). We also investigated the subpopulations of all the 52 asthmatic patients using PAM method based on JSD distance and PCA analysis, we identified five clusters (types) in our patients. Type 1 had a relatively high level of genus Wallemia, type 2, a high level of Candida, type 3 was made up of highly abundant Alternaria, type 4 was made up of highly abundant Plectosphaerella, and type 5 was highly enriched in unclassified_k_Fungi. In addition, type 1, 2, 3, 5 was consisted of both naïve patients and ICS receiving patients, however, type 4 consisted only ICS receiving patients (Additional file 18: Fig S10).

Airway bacteriome

4,489,868 amplicon sequence reads (average sequence length, 420.5 bp) were yielded after quality control, 38 phyla, 379 family, 750 genera, 1349 species and 2096 OUTs were identified for further analysis. Rarefaction curves (Shannon and Simpson indices) on OTU level indicated a large enough sequencing data volume is large enough to cover the microbial information in all samples (Additional file 19: Fig S11).

We also group all the 56 patients into two clusters using PAM method, two types were identified. Type 1 had a relatively high level of genus Streptococcus, type 2, a high level of Neisseria. The two types were consisted of both native patients and ICS receiving patients (Additional file 20: Fig S12).

Reference

1. Kuczynski J, Stombaugh J, Walters WA, González A, Caporaso JG, Knight R. Using QIIME to analyze 16S rRNA gene sequences from microbial communities. Curr Protoc Bioinformatics. 2011;Chapter 10:Unit 10.7.

2. Turnbaugh PJ, Hamady M, Yatsunenko T, Cantarel BL, Duncan A, Ley RE, et al. A core gut microbiome in obese and lean twins. Nature. 2009;457:480–4.

3. Krzywinski M, Schein J, Birol I, Connors J, Gascoyne R, Horsman D, et al. Circos: an information aesthetic for comparative genomics. Genome Res. 2009;19:1639–45.

4. Arumugam M, Raes J, Pelletier E, Le Paslier D, Yamada T, Mende DR, et al. Enterotypes of the human gut microbiome. Nature. 2011;473:174–80.

5. Blankenberg D, Von Kuster G, Coraor N, Ananda G, Lazarus R, Mangan M, et al. Galaxy: a web-based genome analysis tool for experimentalists. Curr Protoc Mol Biol. 2010;Chapter 19:Unit 19.10.1-21.
