# Supplementary material for: Fungal and bacterial microbiome dysbiosis and imbalance of trans-kingdom network in asthma
Source: Clin Transl Allergy. 2020 Oct 22;10:42. doi: 10.1186/s13601-020-00345-8 (PMC7583303; doi:10.1186/s13601-020-00345-8)
Supplement: Supplementary file 2 — Additional file 2: Table S1. Demographic and clinical characteristics of study subjects (mycobiome) (n=68). [file 13601_2020_345_MOESM2_ESM.pdf]

Additional file 2. Table S1. Demographic and clinical characteristics of study subjects (mycobiome) (n=68)

Variables	**CON (n=16)**	**Untreated asthma (n=22)**	**ICS asthma (n=30)**
Age, years, mean ± SD	38.2 ± 9.6	42.45 ± 14.8	44.86 ± 15.7
Male, no. (%)	6(37.5)	9(40.9)	11(36.6%)
BMI, kg/m2, mean ± SD	22.2 ± 2.9	24.2 ± 3.9	22.7 ± 3.0
Duration of asthma, years, mean ± SD)	-	2.3 ± 6.5	10.8 ± 16.6###
Rhinosinusitis, no. (%)	0(0)	15(68.1) ***	17(56.6)
ICS dose[1], µg.day-1, mean ± SD	-	-	569.6 ± 302.9
FEV1 (% predicted), mean ± SD	95 ± 9.5	72.1 ± 22.6***	82.8 ± 17.6
FEV1/FVC (%), mean ± SD	82.5 ± 7.7	70.1 ± 10.3**	75.2 ± 12.9
ACQ7 score, mean ± SD	-	1.3 ± 0.8	0.7 ± 0.5##
Sputum eosinophils (EOS%), mean ± SD	1 ± 1.3	2.9 ± 2.7	2.0 ± 2.5
Sputum neutrophils (NEU%), mean ± SD	27.3 ± 23.7	21.8 ± 19.5	17.4 ± 16.0
Total IgE, IU/mL, mean ± SD	44.1 ± 16.6	157.6 ± 117.0 ***	155.2 ± 72.6

[1] ICS dose was expressed as beclomethasone propionate equivalent dose. Naïve asthma group vs CON group: **p<0.01, ***p<0.001. ICS asthma group vs naïve asthma group: ## p<0.01 ### p<0.001.
