# Supplementary material for: Fungal and bacterial microbiome dysbiosis and imbalance of trans-kingdom network in asthma
Source: Clin Transl Allergy. 2020 Oct 22;10:42. doi: 10.1186/s13601-020-00345-8 (PMC7583303; doi:10.1186/s13601-020-00345-8)

Additional file 3. Fig. S1. Relationships between variability in airway mycobiome composition and community diversity. a and b. Increased richness (higher Ace and Chao indices) is correlated with higher FEV1%pre. c. Increased phylogenetic diversity (higher PD index) is correlated with higher FEV1%pre. d. Increased richness (higher Chao index) is correlated with lower ACQ 7 score.

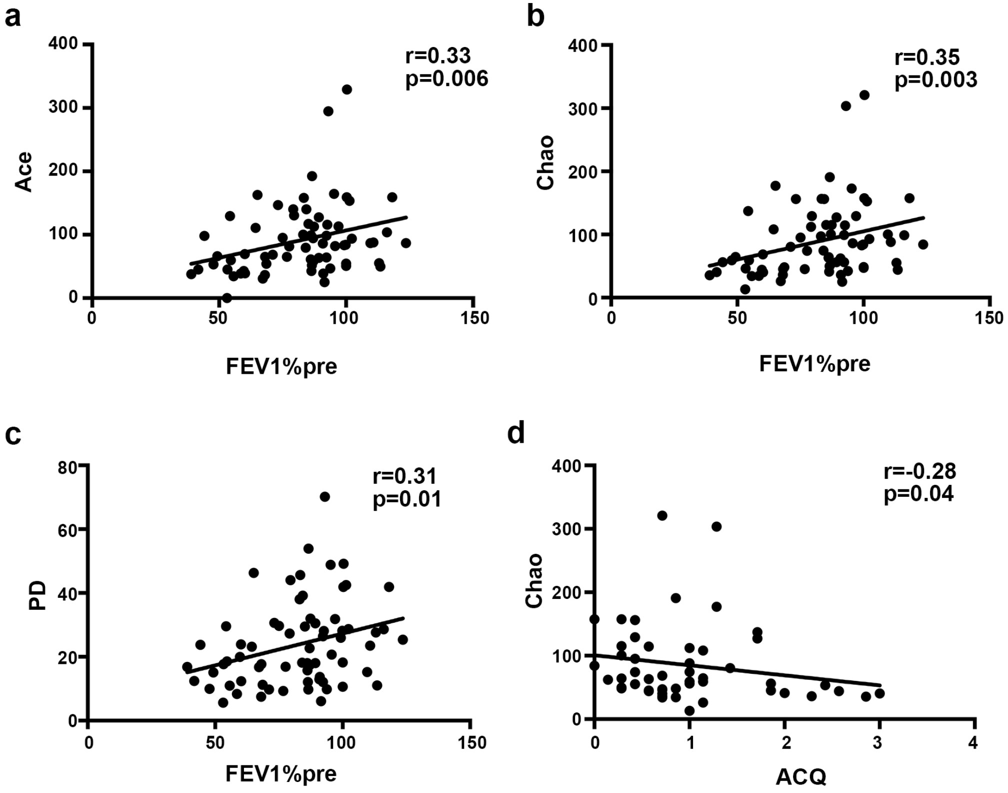

Supplement: Supplementary file 3 — Additional file 3: Fig. S1. Relationships between variability in airway mycobiome composition and community diversity. a and b. Increased richness (higher Aceand Chao indices) is correlated with higher FEV1%pre. c. Increased phylogenetic diversity (higher PD index) is correlated with higher FEV1%pre.d. Increased richness (higher Chao index) is correlated with lower ACQ 7 score. [file 13601_2020_345_MOESM3_ESM.pdf]
