# Supplementary material for: Fungal and bacterial microbiome dysbiosis and imbalance of trans-kingdom network in asthma
Source: Clin Transl Allergy. 2020 Oct 22;10:42. doi: 10.1186/s13601-020-00345-8 (PMC7583303; doi:10.1186/s13601-020-00345-8)
Supplement: Supplementary file 4 — Additional file 4: Table S2. PERMANOVA of mycobiome community composition in sputum based on Bray-Curtis distance. [file 13601_2020_345_MOESM4_ESM.pdf]

Additional file 4. Table S2. PERMANOVA of mycobiome community composition in sputum based on Bray-Curtis distance.

Matrix	SS	MS	F	R2	P value
Bray-Curtis	2.23878	1.11939	3.79069	0.10445	0.001
