# Supplementary material for: Fungal and bacterial microbiome dysbiosis and imbalance of trans-kingdom network in asthma
Source: Clin Transl Allergy. 2020 Oct 22;10:42. doi: 10.1186/s13601-020-00345-8 (PMC7583303; doi:10.1186/s13601-020-00345-8)
Supplement: Supplementary file 6 — Additional file 6: Table S3. Relative abundance of top 15 genera in airway mycobiome differing significantly between CON and untreated asthma group. [file 13601_2020_345_MOESM6_ESM.pdf]

Additional file 6. Table S3. Relative abundance of top 15 genera in airway mycobiome differing significantly between CON and untreated asthma group.

Phylum	Class	Order	Family	Genus	More abundant (Untreated vs CON)
Basidiomycota	Wallemiomycetes	Wallemiales	Wallemiaceae	Wallemia	Untreated asthma
	Microbotryomycetes	Sporidiobolales	Sporidiobolaceae	Rhodotorula	Untreated asthma
				Sporobolomyces	CON
	Agaricomycetes	Polyporales	Meruliaceae	Irpex	Untreated asthma
			Coriolaceae	Trametes	CON
		Agaricales	Schizophyllaceae	Schizophyllum	Untreated asthma
	Tremellomycetes	Filobasidiales	Filobasidiaceae	Naganishia	CON
Mortierellomycota	Mortierellomycetes	Mortierellales	Mortierellaceae	Mortierella	Untreated asthma
Ascomycota	Leotiomycetes	Helotiales	Sclerotiniaceae	unclassified_f__Sclerotiniaceae	CON
	Sordariomycetes	Hypocreales	Nectriaceae	Fusarium	Untreated asthma
			Clavicipitaceae	Metarhizium	Untreated asthma
		Sordariales	Chaetomiaceae	unclassified_f__Chaetomiaceae	Untreated asthma
	Dothideomycetes	Capnodiales	Mycosphaerellaceae	Mycosphaerella	CON
		Pleosporales	Sporormiaceae	unclassified_f__Sporormiaceae	Untreated asthma
	Eurotiomycetes	Chaetothyriales	Herpotrichiellaceae	Phialophora	Untreated asthma
