# Supplementary material for: Fungal and bacterial microbiome dysbiosis and imbalance of trans-kingdom network in asthma
Source: Clin Transl Allergy. 2020 Oct 22;10:42. doi: 10.1186/s13601-020-00345-8 (PMC7583303; doi:10.1186/s13601-020-00345-8)
Supplement: Supplementary file 7 — Additional file 7: Table S4. Relative abundance of top 15 genera in airway mycobiome differing significantly between untreated asthma and ICS asthma group. [file 13601_2020_345_MOESM7_ESM.pdf]

Additional file 7. Table S4. Relative abundance of top 15 genera in airway mycobiome differing significantly between untreated asthma and ICS asthma groups.

Phylum	Class	Order	Family	Genus	More abundant (ICS vs Untreated)
Basidiomycota	Wallemiomycetes	Wallemiales	Wallemiaceae	Wallemia	Untreated asthma
	Tremellomycetes	Cystofilobasidiales	Cystofilobasidiaceae	Guehomyces	Untreated asthma
	Agaricomycetes	Agaricales	Psathyrellaceae	Coprinellus	Untreated asthma
		Cantharellales	Cantharellales_fam_Incertae_sedis	Sistotrema	ICS asthma
	Microbotryomycetes	Sporidiobolales	Sporidiobolaceae	Sporobolomyces	Untreated asthma
Ascomycota	Dothideomycetes	Pleosporales	Pleosporaceae	Alternaria	Untreated asthma
			Sporormiaceae	unclassified_f__Sporormiaceae	ICS asthma
	Eurotiomycetes	Eurotiales	Aspergillaceae	Aspergillus	Untreated asthma
		Chaetothyriales	Herpotrichiellaceae	Phialophora	ICS asthma
	Sordariomycetes	Hypocreales	Nectriaceae	Fusarium	ICS asthma
		Xylariales	Xylariales_fam_Incertae_sedis	Phialemoniopsis	ICS asthma
		Sordariales	Chaetomiaceae	unclassified_f__Chaetomiaceae	ICS asthma
	Leotiomycetes	Helotiales	Sclerotiniaceae	unclassified_f__Sclerotiniaceae	Untreated asthma

Mortierellomycota	Mortierellomycetes	Mortierellales	Mortierellaceae	Mortierella	ICS asthma
unclassified_k__Fungi	unclassified_k__Fungi	unclassified_k__Fungi	unclassified_k__Fungi	g__unclassified_k__Fungi	ICS asthma
