# Supplementary material for: Fungal and bacterial microbiome dysbiosis and imbalance of trans-kingdom network in asthma
Source: Clin Transl Allergy. 2020 Oct 22;10:42. doi: 10.1186/s13601-020-00345-8 (PMC7583303; doi:10.1186/s13601-020-00345-8)
Supplement: Supplementary file 8 — Additional file 8: Table S5. Demographic and clinical characteristics of study subjects (bacteriome) (n=82) [file 13601_2020_345_MOESM8_ESM.pdf]

Additional file 8. Table S5. Demographic and clinical characteristics of study subjects (bacteriome) (n=82)

Variables	**CON (n=26)**	**Untreated asthma (n=23)**	**ICS asthma (n=33)**
Age, years, mean ± SD	40.9 ± 9.5	44.7 ± 14.9	45.5 ± 15.7
Male, no. (%)	14(53.8)	10(43.4)	11(33.3)
BMI, kg/m2, mean ± SD	22.9 ± 3.1	23.8 ± 4.1	22.6 ± 2.9
Duration of asthma, years, mean ± SD)	-	2.2 ± 6.3	10.7 ± 16.1###
Rhinosinusitis, no. (%)	0(0)	15(65.2) ***	20(60.0)
ICS dose[1], µg.day-1, mean ± SD	-	-	555.6 ± 298.6
FEV1 (% predicted), mean ± SD	94.7 ± 8.8	75.7 ± 23.3**	83.6 ± 17.6###
FEV1/FVC (%), mean ± SD	82.2 ± 6.5	71.5 ± 11.6**	75.3 ± 12.7
ACQ7 score, mean ± SD	-	1.1 ± 0.7	0.7 ± 0.5#
Sputum eosinophils (%), mean ± SD	1.0 ± 1.1	3.0 ± 2.5*	1.9 ± 2.5
Sputum neutrophils (%), mean ± SD	28.6 ± 24.9	21.6 ± 18.9	18.3 ± 16.5
Total IgE, IU/mL, mean ± SD	43.3 ± 15.9	158.1 ± 114.4***	142.0 ± 77.2

[1] ICS dose was expressed as beclomethasone propionate equivalent dose. Naïve asthma group vs CON group: *$p<0.05$, **$p<0.01$, ***$p<0.001$. ICS asthma group vs naïve asthma group: # $p<0.05$, ### $p<0.001$.
