# Supplementary material for: Fungal and bacterial microbiome dysbiosis and imbalance of trans-kingdom network in asthma
Source: Clin Transl Allergy. 2020 Oct 22;10:42. doi: 10.1186/s13601-020-00345-8 (PMC7583303; doi:10.1186/s13601-020-00345-8)
Supplement: Supplementary file 9 — Additional file 9: Table S6. PERMANOVA of bacteriome community composition in sputum based on Bray-Curtis distance. [file 13601_2020_345_MOESM9_ESM.pdf]

Additional file 9. Table S6. PERMANOVA of bacteriome community composition in sputum based on Bray-Curtis distance.

Matrix	SS	MS	F	R2	P value
Bray-Curtis	0.41598	0.20799	1.838839	0.04343	0.016
