# Supplementary material for: Fungal and bacterial microbiome dysbiosis and imbalance of trans-kingdom network in asthma
Source: Clin Transl Allergy. 2020 Oct 22;10:42. doi: 10.1186/s13601-020-00345-8 (PMC7583303; doi:10.1186/s13601-020-00345-8)

Additional file 10. Fig. S3. Circular representation of bacteriome communities in CON, naïve asthma and ICS asthma groups at phylum and genus level, visualized by Circos software. The outmost circles list names of three groups and bacteriome community composition. The length of the bars on the second ring represented the percentage of phyla or genera in three groups (left side of the diagram), and the percentage of sample in phyla or genus. The connecting lines inside the third circle link the phylum or genus to the groups and the width of the line indicates the relative abundance. The outset cycles were coloured according to the software default setting.

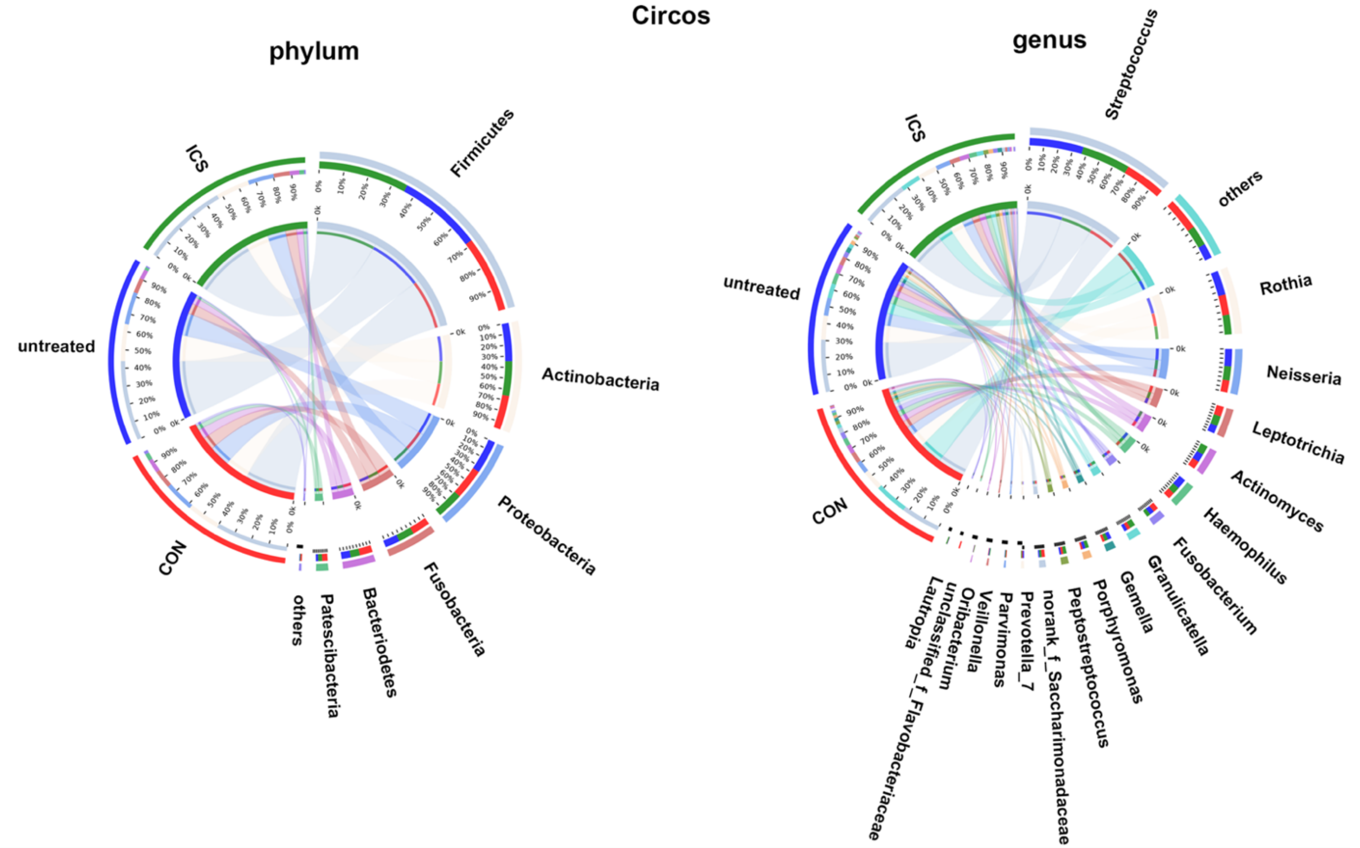

Supplement: Supplementary file 10 — Additional file 10: Fig. S3. Circular representation of bacteriome communities in CON, naïve asthma and ICS asthma groups at phylum and genus level, visualized by Circos software. The outmost circles list names of three groups and bacteriome community composition. The length of the bars on the secondring represented the percentage of phyla or genera in three groups (left side of the diagram), and the percentage of sample in phyla or genus. The connecting lines inside the third circle link the phylum or genus to the groups and the width of the line indicates the relative abundance. The outset cycles were coloured according to the software default setting. [file 13601_2020_345_MOESM10_ESM.pdf]
