# Supplementary material for: Fungal and bacterial microbiome dysbiosis and imbalance of trans-kingdom network in asthma
Source: Clin Transl Allergy. 2020 Oct 22;10:42. doi: 10.1186/s13601-020-00345-8 (PMC7583303; doi:10.1186/s13601-020-00345-8)
Supplement: Supplementary file 11 — Additional file 11: Fig. S4. Relative abundance of top 15 genera in airway bacteriome differing significantly between CON and untreated asthma group (a), between untreated asthma group and ICS asthma group (b). Statistical significance was determined using Kruskal-Wallis rank-sum test. [file 13601_2020_345_MOESM11_ESM.pdf]

Additional file 11. Fig. S4. Relative abundance of top 15 genera in airway bacteriome differing significantly between CON and untreated asthma group (a), between untreated asthma group and ICS asthma group (b). Statistical significance was determined using Kruskal-Wallis rank-sum test.

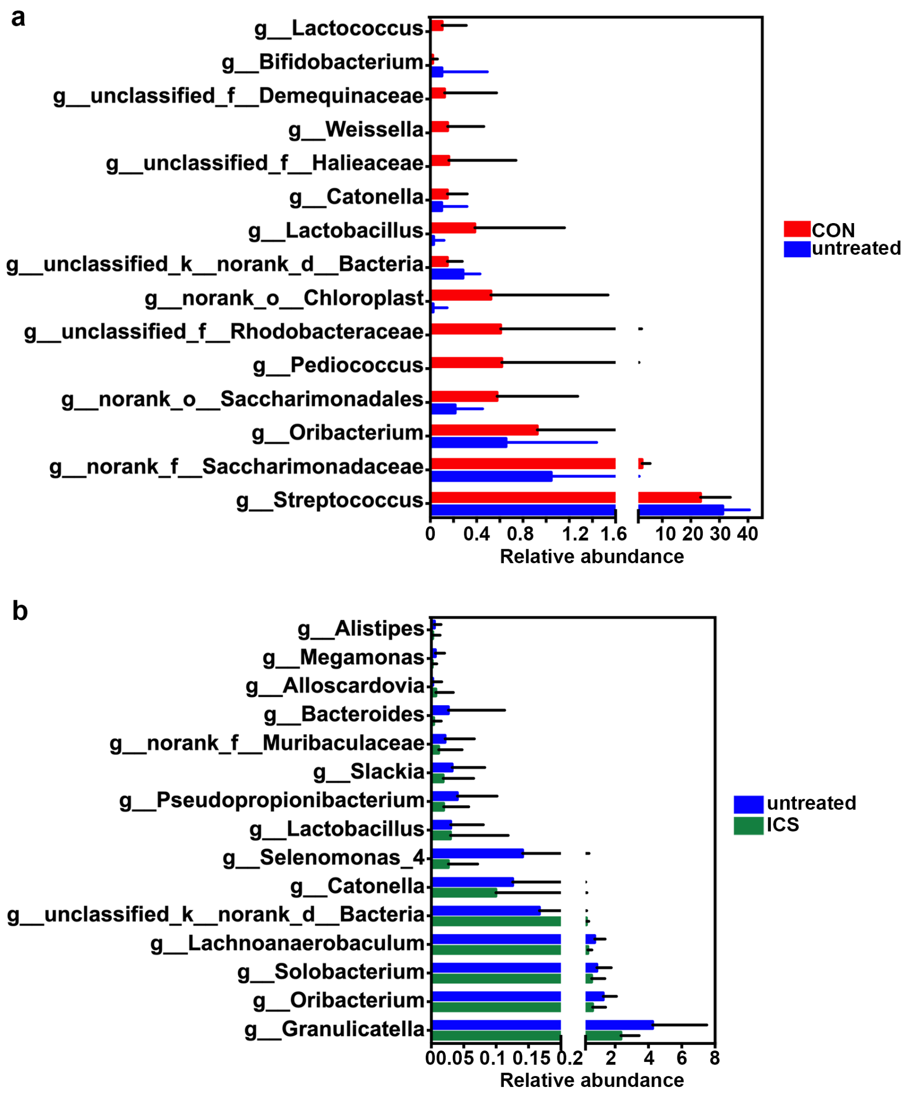
a
g__Lactococcus
g__Bifidobacterium
g__unclassified_f__Demequinaceae
g__Weissella
g__unclassified_f__Halieaceae
g__Catonella
g__Lactobacillus
g__unclassified_k__norank_d__Bacteria
g__norank_o__Chloroplast
g__unclassified_f__Rhodobacteraceae
g__Pediococcus
g__norank_o__Saccharimonadales
g__Oribacterium
g__norank_f__Saccharimonadaceae
g__Streptococcus
0 0.4 0.8 1.2 1.6 10 20 30 40
Relative abundance
CON
untreated
b
g__Alistipes
g__Megamonas
g__Alloscardovia
g__Bacteroides
g__norank_f__Muribaculaceae
g__Slackia
g__Pseudopropionibacterium
g__Lactobacillus
g__Selenomonas_4
g__Catonella
g__unclassified_k__norank_d__Bacteria
g__Lachnoanaerobaculum
g__Solobacterium
g__Oribacterium
g__Granulicatella
0 0.05 0.1 0.15 0.2 2 4 6 8
Relative abundance
untreated
ICS

3
