# Supplementary material for: Fungal and bacterial microbiome dysbiosis and imbalance of trans-kingdom network in asthma
Source: Clin Transl Allergy. 2020 Oct 22;10:42. doi: 10.1186/s13601-020-00345-8 (PMC7583303; doi:10.1186/s13601-020-00345-8)
Supplement: Supplementary file 12 — Additional file 12: Table S7. Relative abundance of top 15 genera in airway bacteriome differing significantly between CON and untreated asthma group. [file 13601_2020_345_MOESM12_ESM.pdf]

Additional file 12. Table S7. Relative abundance of top 15 genera in airway bacteriome differing significantly between CON and untreated asthma group.

Gram stain	Phylum	Class	Order	Family	Genus	More abundant (Untreated vs CON)
Positive	Firmicutes	Bacilli	Lactobacillales	Streptococcaceae	Streptococcus	Untreated asthma
					Lactococcus	CON
				Lactobacillaceae	Pediococcus	CON
					Lactobacillus	CON
				Leuconostocaceae	Weissella	CON
		Clostridia	Clostridiales	Lachnospiraceae	Oribacterium	CON
					Catonella	CON
	Actinobacteria	Actinobacteria	Micrococcales	Demequinaceae	g__unclassified_f__Demequinaceae	CON
			Bifidobacteriales	Bifidobacteriaceae	Bifidobacterium	Untreated asthma
-	Patescibacteria	Saccharimonadia	Saccharimonadales	Saccharimonadaceae	norank_f_Saccharimonadaceae	CON
				norank_o__Saccharimonadales	norank_o__Saccharimonadales	CON
Negative	Proteobacteria	Alphaproteobacteria	Rhodobacterales	Rhodobacteraceae	unclassified_f__Rhodobacteraceae	CON
		Gammaproteobacteria	Cellvibrionales	Halieaceae	unclassified_f__Halieaceae	CON
	Cyanobacteria	Oxyphotobacteria	Chloroplast	norank_o__Chloroplast	norank_o__Chloroplast	CON

-	unclassified_k__norank_d__Bacteria	unclassified_k__norank_d__Bacteria	unclassified_k__norank_d__Bacteria	unclassified_k__norank_d__Bacteria	unclassified_k__norank_d__Bacteria	Untreated asthma
