# Supplementary material for: Fungal and bacterial microbiome dysbiosis and imbalance of trans-kingdom network in asthma
Source: Clin Transl Allergy. 2020 Oct 22;10:42. doi: 10.1186/s13601-020-00345-8 (PMC7583303; doi:10.1186/s13601-020-00345-8)
Supplement: Supplementary file 13 — Additional file 13: Table S8. Relative abundance of top 15 genera in airway bacteriome differing significantly between untreated asthma and ICS asthma groups. [file 13601_2020_345_MOESM13_ESM.pdf]

Additional file 13. Table S8. Relative abundance of top 15 genera in airway bacteriome differing significantly between untreated asthma and ICS asthma group.

Gram stain	Phylum	Class	Order	Family	Genus	More abundant (ICS vs Untreated)
Positive	Firmicutes	Bacilli	Lactobacillales	Carnobacteriaceae	Granulicatella	Untreated asthma
				Lactobacillaceae	Lactobacillus	Untreated asthma
		Clostridia	Clostridiales	Lachnospiraceae	Oribacterium	Untreated asthma
					Lachnoanaerobaculum	Untreated asthma
					Catonella	Untreated asthma
		Erysipelotrichia	Erysipelotrichales	Erysipelotrichaceae	Solobacterium	Untreated asthma
		Negativicutes	Selenomonadales	Veillonellaceae	Selenomonas_4	Untreated asthma
					Megamonas	Untreated asthma
	Actinobacteria	Actinobacteria	Propionibacteriales	Propionibacteriaceae	Pseudopropionibacterium	Untreated asthma
			Coriobacteriales	Eggerthellaceae	Slackia	Untreated asthma
			Bifidobacteriales	Bifidobacteriaceae	Alloscardovia	ICS asthma
Negative	Bacteroidetes	Bacteroidia	Bacteroidales	Muribaculaceae	norank_f__Muribaculaceae	Untreated asthma
				Bacteroidaceae	Bacteroides	Untreated asthma
				Rikenellaceae	Alistipes	Untreated asthma
-	unclassified_k__norank_d__Bacteria	unclassified_k__norank_d__Bacteria	unclassified_k__norank_d__Bacteria	unclassified_k__norank_d__Bacteria	unclassified_k__norank_d__Bacteria	ICS asthma
