# Supplementary material for: Fungal and bacterial microbiome dysbiosis and imbalance of trans-kingdom network in asthma
Source: Clin Transl Allergy. 2020 Oct 22;10:42. doi: 10.1186/s13601-020-00345-8 (PMC7583303; doi:10.1186/s13601-020-00345-8)
Supplement: Supplementary file 14 — Additional file 14: Fig. S5. Prediction models and discriminant taxa of bacteriome. a. Prediction models using Random Forest (RF). X-axis represents the number of important species (variables) ranking top n, y-axis represents the corresponding prediction error rate using 10-fold cross validation (CV). b. The Receiver Operating Characteristic (ROC) curve for the random forest model. c,d. LEfSe analysis showing the differentially abundant taxonomy(CON vs untreated asthma group, untreated asthma group vs ICS asthma group). The LDA scores (log10) > 2 are listed, p ≤ 0.05. [file 13601_2020_345_MOESM14_ESM.pdf]

Additional file 14. Fig. S5. Prediction models and discriminant taxa of bacteriome. a. Prediction models using Random Forest (RF). X-axis represents the number of important species (variables) ranking top n, y-axis represents the corresponding prediction error rate using 10-fold cross validation (CV). b. The Receiver Operating Characteristic (ROC) curve for the random forest model. c,d. LEfSe analysis showing the differentially abundant taxonomy (CON vs untreated asthma group, untreated asthma group vs ICS asthma group). The LDA scores (log10) > 2 are listed, $p \leq 0.05$.

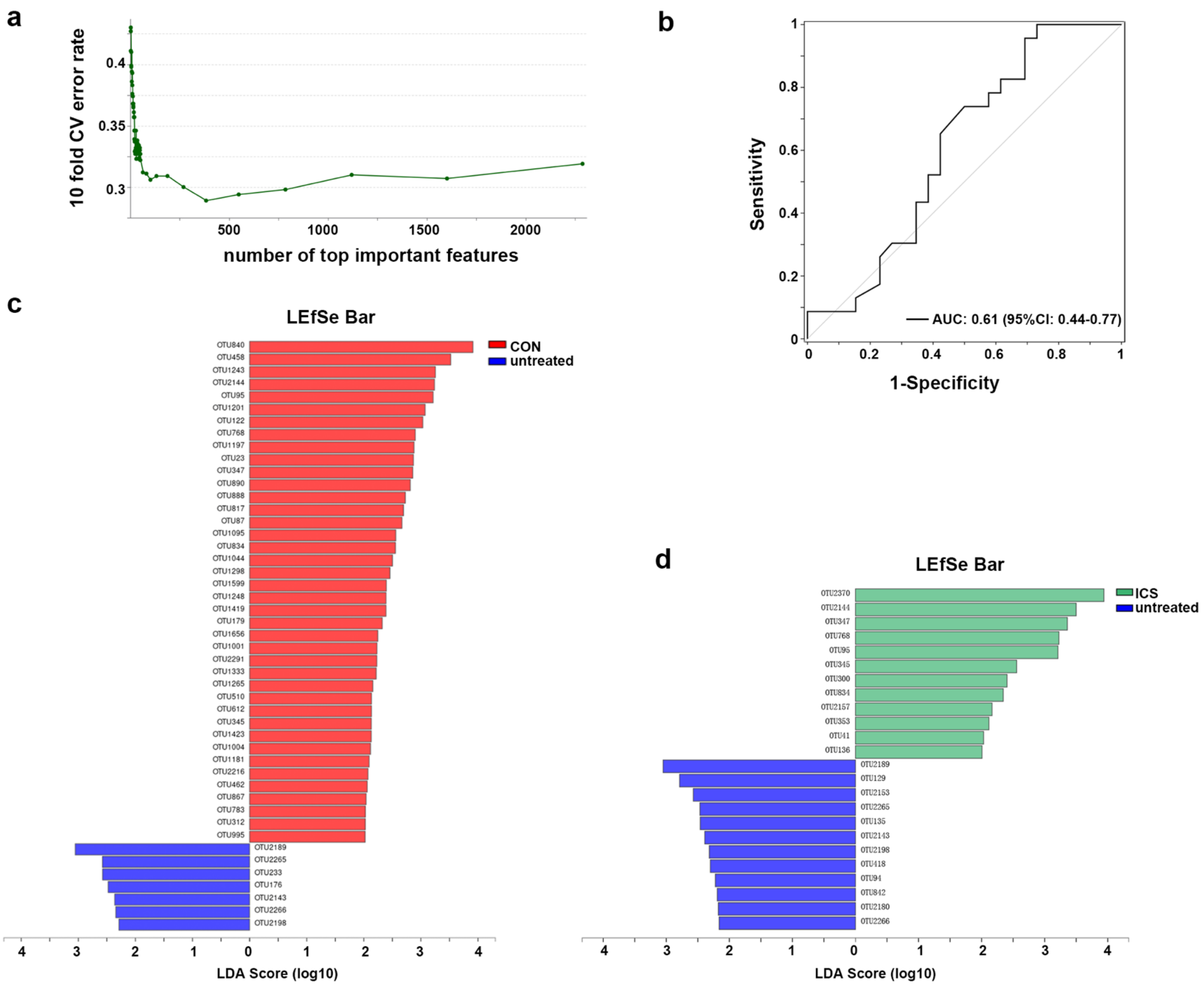
a
10 fold CV error rate
0.4
0.35
0.3
500
1000
1500
2000
number of top important features
b
Sensitivity
1-Specificity
AUC: 0.61 (95%CI: 0.44-0.77)
c
LEfSe Bar
CON
untreated
OTU840
OTU458
OTU1243
OTU2144
OTU95
OTU1201
OTU122
OTU768
OTU1197
OTU23
OTU347
OTU890
OTU888
OTU817
OTU87
OTU1095
OTU834
OTU1044
OTU1298
OTU1599
OTU1248
OTU1419
OTU179
OTU1656
OTU1001
OTU2291
OTU1333
OTU1265
OTU510
OTU612
OTU345
OTU1423
OTU1004
OTU1181
OTU2216
OTU462
OTU867
OTU783
OTU312
OTU995
OTU2189
OTU2265
OTU233
OTU176
OTU2143
OTU2266
OTU2198
LDA Score (log10)
d
LEfSe Bar
ICS
untreated
OTU2370
OTU2144
OTU347
OTU768
OTU95
OTU345
OTU300
OTU834
OTU2157
OTU353
OTU41
OTU136
OTU2189
OTU129
OTU2153
OTU2265
OTU135
OTU2143
OTU2198
OTU418
OTU94
OTU842
OTU2180
OTU2266
LDA Score (log10)
