# Supplementary material for: Fungal and bacterial microbiome dysbiosis and imbalance of trans-kingdom network in asthma
Source: Clin Transl Allergy. 2020 Oct 22;10:42. doi: 10.1186/s13601-020-00345-8 (PMC7583303; doi:10.1186/s13601-020-00345-8)
Supplement: Supplementary file 15 — Additional file 15: Fig. S6. Comparisons of functional genes between CON and naïve asthma group. a. Functional genes of level 1 (human disease) with significant differences between CON and untreated asthma group were showed (Kruskal-Wallis rank-sum test), corrections were made using the False Discovery Rate multiple testing correction. Results were considered statistically significant for p-values ≤ 0.05. b. Functional genes of level 3 with significant differences between CON and untreated asthma group were showed (Kruskal-Wallis rank-sum test), corrections were made using the False Discovery Rate multiple testing correction. Results were considered statistically significant for p-values ≤ 0.05. [file 13601_2020_345_MOESM15_ESM.pdf]

Additional file 15. Fig. S6. Comparisons of functional genes between CON and naïve asthma group. a. Functional genes of level 1 (human disease) with significant differences between CON and untreated asthma group were showed (Kruskal-Wallis rank-sum test), corrections were made using the False Discovery Rate multiple testing correction. Results were considered statistically significant for p-values ≤ 0.05. b. Functional genes of level 3 with significant differences between CON and untreated asthma group were showed (Kruskal-Wallis rank-sum test), corrections were made using the False Discovery Rate multiple testing correction. Results were considered statistically significant for p-values ≤ 0.05.

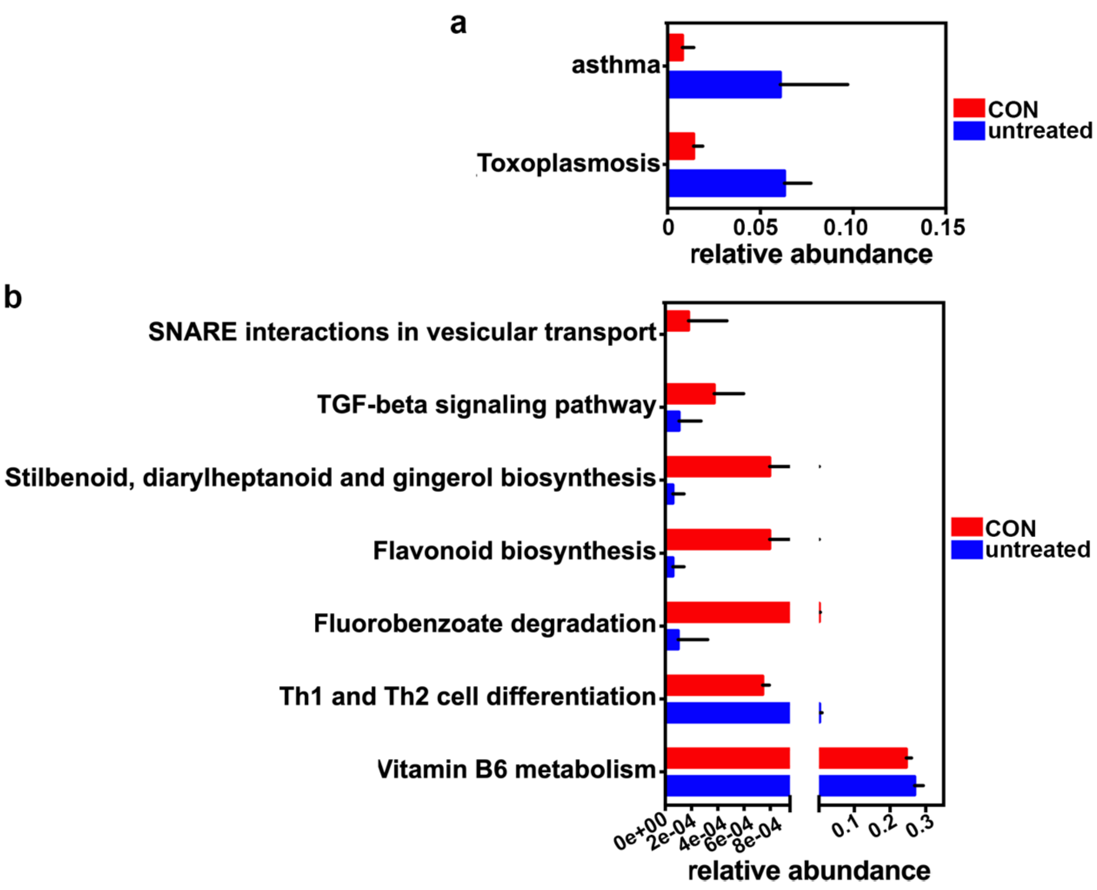

a
asthma
Toxoplasmosis
0
0.05
0.10
0.15
relative abundance
CON
untreated
b
SNARE interactions in vesicular transport
TGF-beta signaling pathway
Stilbenoid, diarylheptanoid and gingerol biosynthesis
Flavonoid biosynthesis
Fluorobenzoate degradation
Th1 and Th2 cell differentiation
Vitamin B6 metabolism
0e+00
2e-04
4e-04
6e-04
8e-04
0.1
0.2
0.3
relative abundance
CON
untreated

6
