# Supplementary figures and images for: Fungal and bacterial microbiome dysbiosis and imbalance of trans-kingdom network in asthma
Source: Clin Transl Allergy. 2020 Oct 22;10:42. doi: 10.1186/s13601-020-00345-8 (PMC7583303; doi:10.1186/s13601-020-00345-8)

1 Additional file 16. Fig. S7. Contributed species to Vitamin B6 metabolism.

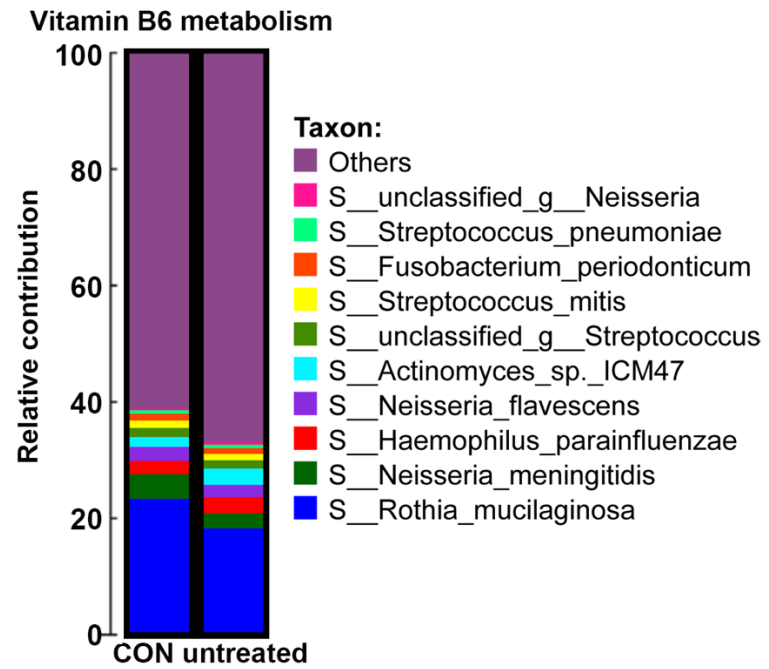

2

Supplement: Supplementary file 16 — Additional file 16: Fig. S7. Contributed species to Vitamin B6 metabolism. [file 13601_2020_345_MOESM16_ESM.pdf]
