# Supplementary material for: Fungal and bacterial microbiome dysbiosis and imbalance of trans-kingdom network in asthma
Source: Clin Transl Allergy. 2020 Oct 22;10:42. doi: 10.1186/s13601-020-00345-8 (PMC7583303; doi:10.1186/s13601-020-00345-8)

Additional file 17. Fig. S8. Correlation network between microbiome and functional genes in CON (a) and untreated (b) group. The node represented a microbial genus, its colour represents the bacterial phylum it belongs to and its size represents the number of direct edges that it has. The green edges indicated positive correlation and red edges indicated negative correlation (Spearman test). Only significant correlations (p value <0.05 after false discovery rate correction) are displayed.

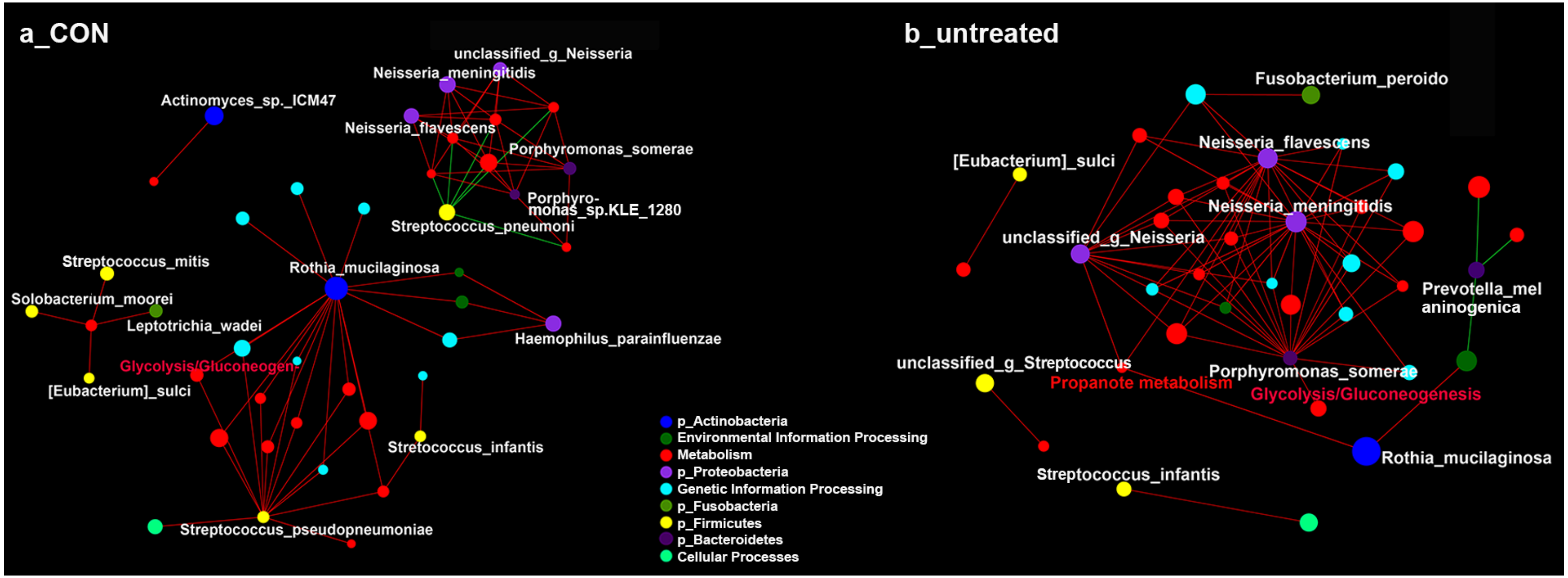

Supplement: Supplementary file 17 — Additional file 17: Fig. S8. Correlation network between microbiome and functional genes in CON (a) and untreated (b) group. The node represented a microbial genus, its colour represents the bacterial phylum it belongs to and its size represents the number of direct edges that it has. The green edges indicated positive correlation and red edges indicated negative correlation (Spearman test). Only significant correlations (p value < 0.05 after false discovery rate correction) are displayed. [file 13601_2020_345_MOESM17_ESM.pdf]
