# Supplementary material for: Fungal and bacterial microbiome dysbiosis and imbalance of trans-kingdom network in asthma
Source: Clin Transl Allergy. 2020 Oct 22;10:42. doi: 10.1186/s13601-020-00345-8 (PMC7583303; doi:10.1186/s13601-020-00345-8)

Additional file 18. Fig. S9. Rarefaction curves of Shannon (a) and Simpson (b) indices of 68 samples (mycobiome). X: number of sequences per sample, Y: rarefaction measure.

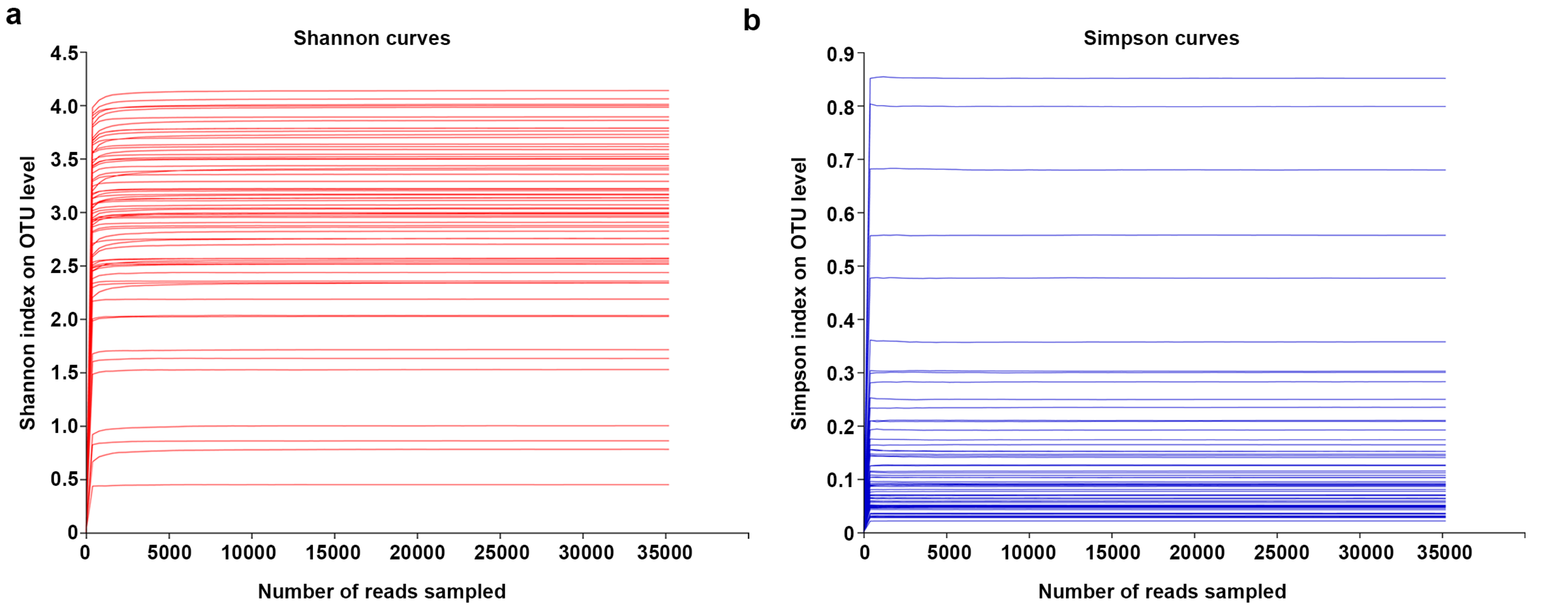

Supplement: Supplementary file 18 — Additional file 18: Fig. S9. Rarefaction curves of Shannon (a) and Simpson (b) indices of 68 samples (mycobiome). X: number of sequences per sample, Y: rarefaction measure. [file 13601_2020_345_MOESM18_ESM.pdf]
