# Supplementary material for: Fungal and bacterial microbiome dysbiosis and imbalance of trans-kingdom network in asthma
Source: Clin Transl Allergy. 2020 Oct 22;10:42. doi: 10.1186/s13601-020-00345-8 (PMC7583303; doi:10.1186/s13601-020-00345-8)
Supplement: Supplementary file 19 — Additional file 19: Fig. S10. Clusters of all the 52 asthmatic patients (mycobiome). a. The optimal number of types was five as indicated by the maximum CH index.b. The airway mycobiome of untreated asthma and ICS asthma groups are clustered into five types at the genus level, dominated by Wallemia (type 1), Candida (type 2), Alternaria (type 3), Plectosphaerella (type 4), and unclassified_k_Fungi (type 5), respectively. c. Relative abundancesof the top 10 genera in the five types (all p < 0.05). d. Distribution of the samples of the two groups in the five types. [file 13601_2020_345_MOESM19_ESM.pdf]

Additional file 19. Fig. S10. Clusters of all the 52 asthmatic patients (mycobiome). a. The optimal number of types was five as indicated by the maximum CH index. b. The airway mycobiome of untreated asthma and ICS asthma groups are clustered into five types at the genus level, dominated by Wallemia (type 1), Candida (type 2), Alternaria (type 3), Plectosphaerella (type 4), and unclassified_k_Fungi (type 5), respectively. c. Relative abundances of the top 10 genera in the five types (all $p<0.05$). d. Distribution of the samples of the two groups in the five types.

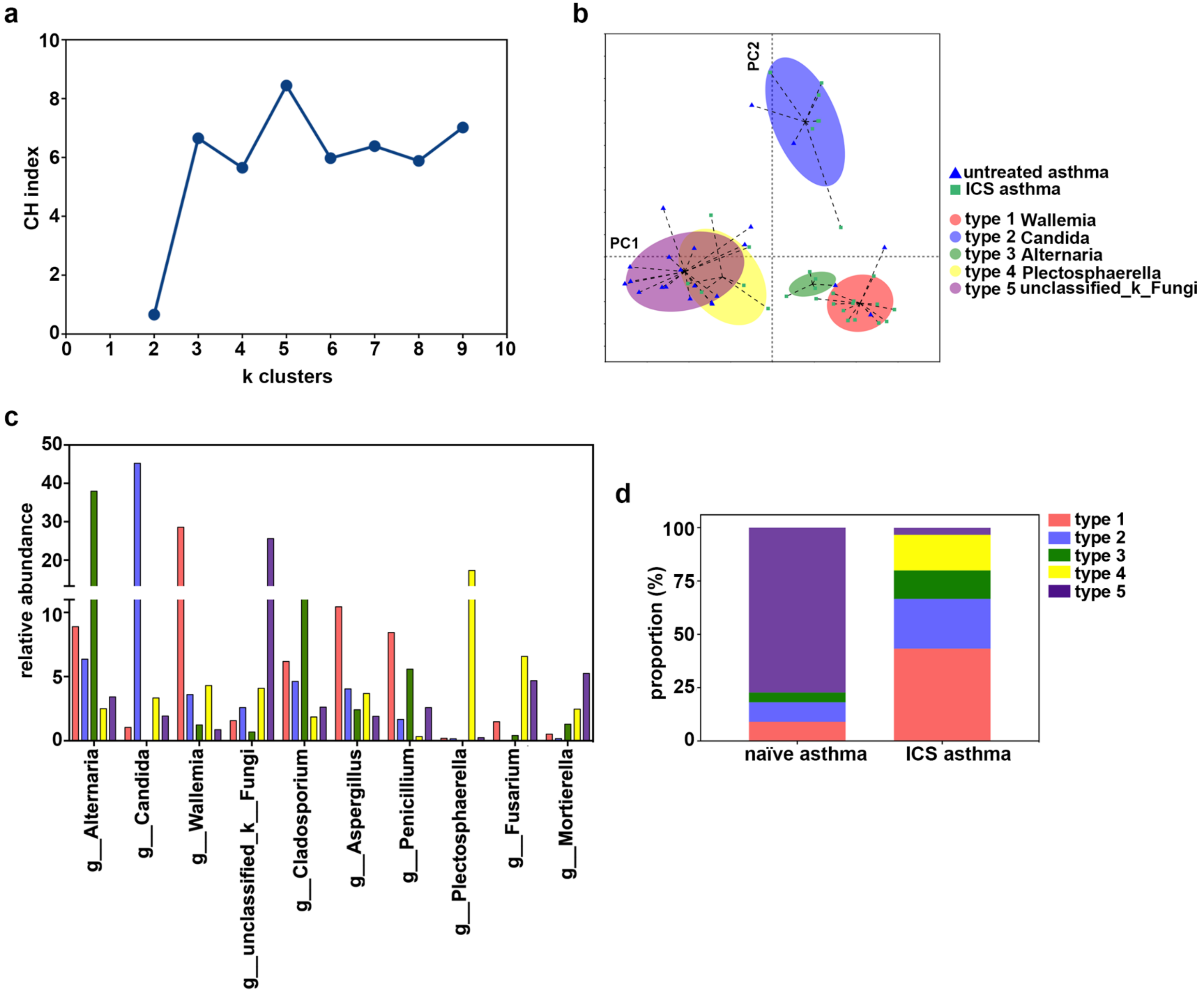
a
CH index
k clusters
b
PC1
PC2
untreated asthma
ICS asthma
type 1 Wallemia
type 2 Candida
type 3 Alternaria
type 4 Plectosphaerella
type 5 unclassified_k_Fungi
c
relative abundance
g__Alternaria
g__Candida
g__Wallemia
g__unclassified_k__Fungi
g__Cladosporium
g__Aspergillus
g__Penicillium
g__Plectosphaerella
g__Fusarium
g__Mortierella
d
proportion (%)
type 1
type 2
type 3
type 4
type 5
naïve asthma
ICS asthma
