# Supplementary material for: Fungal and bacterial microbiome dysbiosis and imbalance of trans-kingdom network in asthma
Source: Clin Transl Allergy. 2020 Oct 22;10:42. doi: 10.1186/s13601-020-00345-8 (PMC7583303; doi:10.1186/s13601-020-00345-8)
Supplement: Supplementary file 21 — Additional file 21: Fig. S12. Clusters of all the 56 asthmatic patients (bacteriome). a. The maximum CH index at two types indicated the optimal enterotype number. b. The airway bacteriome of untreated asthma and ICS asthma groups are clustered into two types at the genus level, dominated by either Streptococcus (type 1) or Neisseria (type 2). c. Relative abundances of the top 10 genera in the two types. The comparisons of Streptococcus, Neisseria, Haemophilus, Porphyromonas and Gemella were statistically different (p < 0.05). d. Distribution of the samples of the four groups in the two types. [file 13601_2020_345_MOESM21_ESM.pdf]

Additional file 21. Fig. S12. Clusters of all the 56 asthmatic patients (bacteriome). a. The maximum CH index at two types indicated the optimal enterotype number. b. The airway bacteriome of untreated asthma and ICS asthma groups are clustered into two types at the genus level, dominated by either Streptococcus (type 1) or Neisseria (type 2). c. Relative abundances of the top 10 genera in the two types. The comparisons of Streptococcus, Neisseria, Haemophilus, Porphyromonas and Gemella were statistically different ($p<0.05$). d. Distribution of the samples of the four groups in the two types.

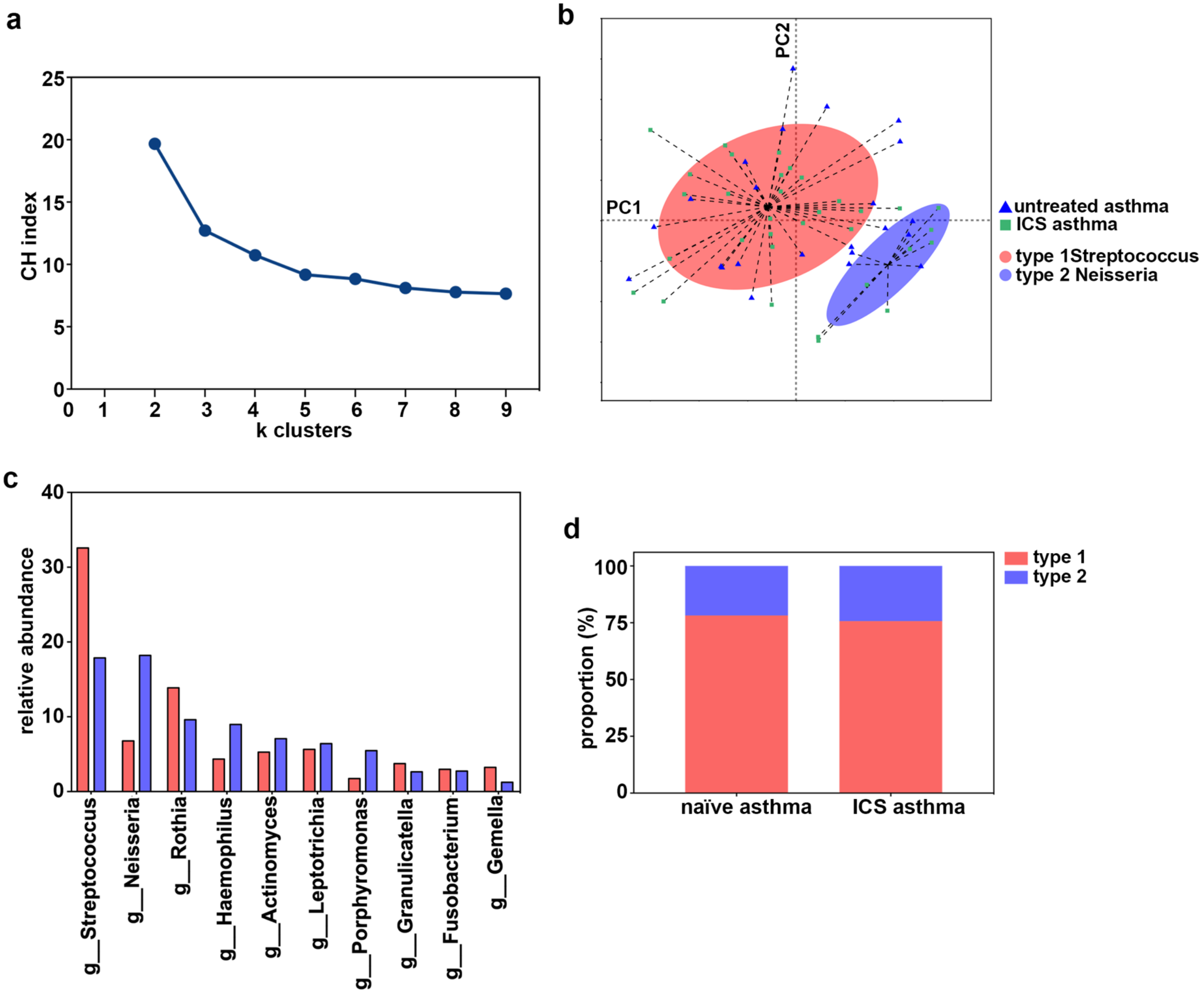
a
CH index
k clusters
0
5
10
15
20
25
0
1
2
3
4
5
6
7
8
9
b
PC1
PC2
untreated asthma
ICS asthma
type 1Streptococcus
type 2 Neisseria
c
relative abundance
0
10
20
30
40
g__Streptococcus
g__Neisseria
g__Rothia
g__Haemophilus
g__Actinomyces
g__Leptotrichia
g__Porphyromonas
g__Granulicatella
g__Fusobacterium
g__Gemella
d
proportion (%)
0
25
50
75
100
naïve asthma
ICS asthma
type 1
type 2
